# Single-Cell Definition and Functional Validation of a CTSD-Associated Macrophage State in Colorectal Cancer

**DOI:** 10.3390/cancers18213464

**Published:** 2026-10-27

**Authors:** Zhao Wang, Bin Hou, Chunyan Liu, Aiguo Meng

**Affiliations:** 1 Affiliated Hospital of North China University of Science and Technology, Tangshan 063000, China; 18231501002@163.com (Z.W.); houbin820211@163.com (B.H.); 2 School of Pharmacy, North China University of Science and Technology, Tangshan 063210, China

**Keywords:** colorectal cancer, tumor-associated macrophages, CTSD, single-cell RNA sequencing, spatial transcriptomics, scissor, tumor–macrophage crosstalk

## Abstract

Background/Objectives: Tumor-associated macrophage heterogeneity may influence colorectal cancer (CRC) outcomes. We investigated a CTSD-associated macrophage program and its functional relevance. Methods: Single-cell GSE132465 data (23 tumor and 10 normal specimens), TCGA colorectal expression and survival data, and three spatial sections were integrated with Scissor, exploratory survival modeling and macrophage-conditioned medium experiments. We added specimen-level pseudobulk expression analysis, including 10 matched tumor–normal pairs, and 100 repeats of stratified ten-fold cross-validation of Cox models in 571 TCGA cases. Results: Of 45,520 cells, a survival-informed macrophage state was identified. CTSD and SPP1 increased in 10/10 matched tumor macrophage specimens (both paired *p* = 0.0020), whereas HSPA1A did not show a significant paired increase (*p* = 0.131). Cross-validated C-indices were 0.550, 0.612 and 0.533 for single-gene CTSD, HSPA1A and SPP1 models, respectively, and 0.604 for the three-gene model. Adding the three genes to age, sex and stage increased the cross-validated C-index from 0.714 to 0.722. Scissor feature selection and downstream survival analyses reused the same outcome, so these results are not independent prognostic validation. CTSD silencing attenuated the ability of newly collected macrophage-conditioned medium to support CRC cell viability, invasion and colony formation. Conclusions: CTSD is a functionally relevant candidate in the experimental macrophage model; clinical prediction and direct extracellular CTSD mechanisms remain unproven.

## Simple Summary

Macrophages can support or restrain colorectal cancer depending on their functional state. Integrated single-cell and TCGA analyses identified a CTSD-associated macrophage program for exploratory study, although Scissor-based selection reused the survival outcome and the optimized state score survival cutoff was not robust to median splitting. New patient-level macrophage pseudobulk analysis of 10 matched tumor–normal pairs supported increased CTSD and SPP1 expression, but not a consistent HSPA1A increase. Repeated ten-fold cross-validation in 571 TCGA cases showed only modest candidate gene discrimination and minimal gain over clinical covariates. Experimentally, CTSD silencing in HT-29-conditioned THP-1-derived macrophage-like cells attenuated the tumor-supporting effects of newly collected macrophage-conditioned medium. These findings support biological candidate prioritization, not an independently validated prognostic signature or proof of direct extracellular CTSD causality.

## 1. Introduction

Colorectal cancer (CRC) remains a major cause of cancer morbidity and mortality worldwide despite advances in screening, surgery and systemic treatment [1,2]. Its biological and clinical heterogeneity reflects both tumor cell alterations and the surrounding immune and stromal ecosystem [3,4,5]. Bulk tissue profiling has improved molecular classification but averages signals across malignant, immune and stromal compartments, limiting resolution of cell state-specific programs.

The immune microenvironment is clinically relevant in CRC because immune cell density, phenotype and spatial distribution are associated with recurrence and treatment response [6,7,8]. Tumor-associated macrophages (TAMs) are abundant and highly plastic. Depending on their state and context, they participate in antigen presentation and antitumor immunity or in angiogenesis, extracellular matrix remodeling, immune suppression, invasion and therapy resistance [9,10,11,12]. This heterogeneity is not captured adequately by total macrophage abundance or a binary M1/M2 classification.

Single-cell RNA sequencing resolves macrophage heterogeneity at cellular resolution and reveals states that are obscured in bulk tissue [13,14,15,16,17]. Scissor links single-cell transcriptional states with patient-level phenotypes, while spatial transcriptomics describes their distribution within tissue [18,19,20,21]. Cell–cell communication and pseudotime analyses characterize ligand–receptor networks and transcriptional continua [22,23,24,25]. These analyses capture complementary features of the tumor microenvironment but do not establish causality.

To examine the functional relevance of the CTSD-associated signal in a macrophage-centered system, we first exposed PMA-differentiated THP-1-derived macrophage-like cells to soluble factors from CRC cell lines. We then silenced CTSD in the HT-29-conditioned macrophage model and assessed the influence of newly collected macrophage-conditioned medium on CRC cell behavior. This experimental sequence tests the contribution of macrophage CTSD perturbation to conditioned medium activity without presuming a canonical M1/M2 state.

We first screened major single-cell compartments for phenotype association and then characterized the macrophage compartment in greater depth. The resulting Mac_Scissor+ state was examined by pathway, communication, pseudotime, bulk immune and spatial analyses. THP-1-derived macrophages were exposed to conditioned media from four CRC cell lines, and CTSD was subsequently silenced in tumor-conditioned macrophages. Macrophage-conditioned media were then tested for effects on CRC cell viability, invasion and colony formation.

## 2. Materials and Methods

### 2.1. Study Design and Data Sources

This study combined public single-cell RNA sequencing, spatial transcriptomics, TCGA bulk transcriptomic and clinical data, and in vitro macrophage tumor-conditioned medium experiments. GSE132465 provided 33 colorectal tissue samples (23 tumor and 10 normal) for single-cell analysis. GSE280313 provided three spatial transcriptomic sections (one tumor and two paired normal sections). The TCGA colorectal cohort included 701 transcriptomic samples (650 tumor and 51 non-tumor); analyses requiring clinical information used the subset with available data. New specimen-level macrophage expression analyses used all 33 GSE132465 specimens, and repeated Cox model cross-validation used 571 TCGA cases with complete survival and clinicopathological data (113 deaths).

### 2.2. Single-Cell RNA-Seq Processing and Cell Type Annotation

Cells expressing fewer than 200 detected genes and cells outside the original analysis thresholds for UMI counts, detected genes or mitochondrial transcript percentage were excluded. Data were normalized with LogNormalize (scale factor 10,000), and 2000 variable genes were used for principal component analysis. Harmony was used for integration, followed by UMAP and graph-based clustering [26,27]. Major cell identities were assigned by canonical lineage markers and SingleR-assisted annotation [28]. Macrophage identification relied on combined C1QA/C1QB, APOC1 and CSF1R expression, with CD68 and ITGAM interpreted alongside, rather than on CTSD alone. Neutrophil-associated S100A8/S100A9 and plasmacytoid dendritic cell markers LILRA4/CLEC4C were inspected to assess overlap; neither individual myeloid markers nor CTSD alone were considered lineage-specific. MRC1, CD163, SPP1 and TREM2 were used to describe within-macrophage state variation. Cluster markers were identified with FindAllMarkers. The epithelial compartment was not subdivided by inferred copy number status; epithelial annotation does not establish malignancy.

### 2.3. Macrophage Subclustering, Scissor Mapping and Survival Analysis

Major single-cell compartments were screened with Scissor [21] using TCGA-COAD/READ overall survival as the patient-level phenotype. Survival time was measured from diagnosis to death from any cause or censoring at last follow-up, with event coded 1 for death and 0 for censoring. Scissor integrated bulk tumor expression, survival outcomes and the integrated single-cell expression profiles, including macrophages from both tumor and normal tissues; positively associated cells were labeled Scissor+, negatively associated cells Scissor−, and unselected cells were assigned to the background category. Thus, Scissor cell selection explicitly used the outcome subsequently examined in the same TCGA cohort. Macrophages were subsetted and reclustered into five unsupervised subclusters, a classification distinct from the Scissor labels. To estimate relative representation of the Scissor+ transcriptional program in TCGA bulk samples, the Mac_Scissor+ marker set was projected onto tumor expression using ssGSEA (GSVA; method = ‘ssgsea’); the resulting enrichment score was treated as a program proxy, not an absolute cellular fraction. The score survival comparison included 517 eligible patients. An optimized cutpoint was selected using survminer::surv_cutpoint (minimum group fraction 0.10); survival separation was also assessed using a fixed median cutoff. Because the marker set was derived from survival-informed cells and the optimal threshold was estimated using the same survival data, the nominal log-rank *p* value cannot be interpreted as independent or fully selection-adjusted prognostic evidence. For descriptive analyses, “other macrophages” combines Scissor− and background/unselected cells; inferential candidate gene DEGs specifically compare Scissor+ against Scissor− macrophages and exclude the background category.

### 2.4. Single-Cell Pathway, Communication and Trajectory Analyses

For single-cell pathway characterization, irGSEA combined AUCell, UCell, singscore and ssGSEA results using robust rank aggregation. Hallmark and, in a supplementary analysis, KEGG pathway gene sets were examined; gene set results were considered exploratory. CellChat inferred putative ligand–receptor signaling from normalized expression and cell annotations [22]. Cell–cell communication scores represent transcript-based predictions, not verified protein interactions; communication rank was not used to select macrophages. CytoTRACE estimated relative differentiation-associated transcriptional potential [23]. Monocle generated a pseudotime ordering [24,25], interpreted as a cross-sectional expression continuum rather than lineage tracing.

### 2.5. Spatial Transcriptomic and Pathway Activity Analyses

The spatial transcriptomic dataset consisted of one colorectal tumor section and two normal-adjacent sections. Following normalization, scaling, PCA and Louvain clustering, CARD estimated reference cell type contributions to spots using single-cell reference counts and annotations [19]. SpaTopic described spatial ecological domains and PROGENy inferred pathway footprints [20]. Mac_Scissor+ abundance was not formally tested against individual CellTopics or PROGENy pathways, and these results were used only for descriptive tissue context. The three-section sample size precludes strong spatial generalization.

### 2.6. Gene Prioritization and Bulk Immune/Pathway Analyses

FindMarkers (Wilcoxon test with Benjamini–Hochberg correction) compared Mac_Scissor+ (*n* = 1912) with Mac_Scissor− macrophages (*n* = 1271); genes with avg_log2FC > 0.25 and adjusted *p* < 0.05 were retained. These cell-level tests did not model patients as replicates; a separate patient/specimen-level pseudobulk analysis was therefore added to evaluate candidate gene expression without cell count pseudoreplication. The resulting 49 genes were mapped directly by gene symbol to corresponding TCGA bulk gene expression features and used as candidate covariates in a randomForestSRC survival forest with 1000 trees [29]. The supplied analysis describes permutation-style out-of-bag variable importance rescaled to the maximum positive importance (=1.0), with an exploratory relative importance selection threshold of >0.20. This operational threshold was not obtained by formal optimization or stability testing and was used for candidate prioritization rather than calibrated prognostic prediction. No nested rerun of Scissor selection, resampling stability analysis of the RSF feature selection procedure or external replication of gene selection was performed; subsequent repeated cross-validation evaluated prespecified candidate gene Cox models only. For individual gene survival analyses, 591 patients with usable bulk expression, positive follow-up and survival outcome information were split at each gene’s median expression (high *n* = 296; low *n* = 295). Univariable log-rank and Cox analyses were performed, including Cox models for continuous expression standardized to one SD. Separate exploratory Cox models adjusted for age, sex and stage in 571 complete cases; concordance indices for single-gene univariable models were computed. As the same TCGA survival endpoint informed Scissor selection and subsequent analyses, the reported gene effects and the new cross-validated concordance estimates remain internal, selection-conditioned findings rather than independent prognostic validation. CIBERSORT LM22 estimated relative immune cell fractions [30], whose M0/M1/M2 labels were not equated with single-cell Scissor states. Bulk KEGG GSEA used expression-ranked lists generated by comparing each key gene’s median split groups (clusterProfiler::gseKEGG; organism = ‘hsa’) [31]; GSVA used the MSigDB Hallmark gene sets and limma to examine differential pathway scores [32,33].

#### 2.6.1. Patient-Level Macrophage Pseudobulk Expression Validation

To address donor-level non-independence, raw UMI counts from all annotated macrophages were summed by gene within each GSE132465 tissue specimen, yielding 33 specimen-level pseudobulk profiles from 23 patients (23 tumor and 10 normal-adjacent specimens; 10 patients contributed paired specimens). Macrophages were pooled regardless of Scissor label, providing a tissue-level expression comparison rather than a survival-specific state test. Expression was normalized to counts per million (CPM) using each specimen’s aggregate macrophage library total, followed by log2(CPM + 1) transformation for visualization. The ten matched tumor–normal pairs were assessed with an exact two-sided Wilcoxon signed rank test; the 23 tumor versus 10 normal specimens were additionally compared using an unpaired Wilcoxon rank sum test (normal approximation with continuity correction for ties). For count-based inference, DESeq2 was fitted on the 20 paired pseudobulk samples using a patient-blocked design (~patient + tissue); two-sided Wald *p* values were adjusted by the Benjamini–Hochberg method. Results are reported for CTSD, HSPA1A and SPP1, including nonsignificant findings (Figure S17 and Table S4).

#### 2.6.2. Repeated Internal Cross-Validation of Candidate Gene Cox Models

The TCGA-COAD/READ complete case subset (*n* = 571; 113 deaths) was used for repeated, event-stratified ten-fold cross-validation of Cox proportional hazards models. We assessed CTSD, HSPA1A and SPP1 individually as continuous per-SD expression covariates; a joint three-gene model; each candidate and the joint model adjusted for age, sex and pathologic stage; an age/sex/stage-only reference model; and three single-gene median split models (12 specifications in total). Each ten-fold partition was randomly regenerated 100 times while approximately balancing death events across folds. Models were fitted only on training folds, and held-out linear predictors were evaluated using Harrell’s concordance index; a pooled held-out C-index was calculated for each repeat. For median split models, the expression cutpoint was estimated within each training fold. We report the apparent full-data C-index, mean and standard deviation of the 100 pooled held-out C-indices, their 2.5th–97.5th percentile distribution across partitions, and the apparent-minus-cross-validated difference. These percentiles describe partition sensitivity, not a 95% confidence interval for future cohort performance. Scissor cell selection and gene prioritization were not nested within the folds; therefore, this procedure evaluates the stability of fixed candidate models, not selection-independent prognostic performance (Figure S18 and Table S5).

### 2.7. Cell Culture and Tumor Cell-Conditioned Medium

Established human CRC cell lines RKO, HCT116, SW480 and HT-29 and the human monocytic cell line THP-1 were used. Cells were maintained at 37 °C in a humidified atmosphere containing 5% CO_2_. CRC cell lines were cultured in complete medium supplemented with 10% fetal bovine serum and 1% penicillin–streptomycin. For tumor cell-conditioned medium, CRC cells at approximately 70–80% confluence were washed and incubated in serum-reduced collection medium for 24 h. Supernatants were collected, centrifuged to remove cellular debris and used immediately or stored at −80 °C.

### 2.8. THP-1 Differentiation and Tumor Cell-Conditioned Medium Exposure

THP-1 monocytes were differentiated into adherent macrophage-like cells with 100 ng/mL phorbol 12-myristate 13-acetate (PMA) for 24 h and allowed to recover in PMA-free medium for 24 h. Differentiated cells were then exposed for 24 h to unconditioned control medium or medium containing 50% conditioned supernatant from RKO, HCT116, SW480 or HT-29 cultures. The baseline control was the PMA-differentiated macrophage-like cell group without CRC-conditioned medium exposure; it was not a normal colon epithelial cell-conditioned medium group. The unconditioned medium was used as the baseline culture control, and identical exposure durations were used. The original record does not establish component-by-component equivalence between this medium and the four CRC collection media. PMA differentiation was used to establish a macrophage-like model, not to designate M1 or M2 polarization. Cells subsequently exposed to HT-29-CM are described as HT-29-conditioned macrophage-like cells.

### 2.9. siRNA-Mediated CTSD Knockdown

After HT-29-CM conditioning, THP-1-derived macrophage-like cells were transfected with non-targeting siRNA (si-NC) or one of three CTSD-targeting siRNAs (50 nM, 48 h). CTSD knockdown was evaluated by qPCR and immunoblotting. The siRNA that produced the greatest reduction in CTSD expression was carried forward for the conditioned medium and recipient CRC cell functional assays. This design directly compares the consequences of CTSD perturbation within the same HT-29-conditioned macrophage-like model.

### 2.10. Quantitative Real-Time PCR

Total RNA was extracted from macrophages and reverse-transcribed to cDNA. qPCR was performed for CTSD, SPP1, HSPA1A and GAPDH. Relative expression was calculated by the 2^−ΔΔCt^ method [34,35]. Each biological sample was measured in technical duplicate; technical pairs differing by more than 0.30 Ct were repeated. ΔCt values were used for inferential statistics.

### 2.11. Western Blotting

Western blotting was performed on lysates of PMA-differentiated THP-1-derived macrophage-like cells; CRC cell lysates were not analyzed. CTSD, SPP1 and HSPA1A signals were normalized to paired GAPDH signals after local background correction. For each biological experiment, the target/GAPDH ratio was calculated; relative abundance was then obtained by dividing this value by the mean target/GAPDH ratio in the corresponding control group. Data are expressed as mean ± SEM from three biological experiments, with one-way ANOVA followed by Dunnett-adjusted comparisons as specified. The unconditioned PMA-differentiated group served as the baseline in the tumor-conditioned medium comparison; si-NC was the baseline for the siRNA knockdown comparison.

### 2.12. ELISA of Macrophage Supernatants

Macrophage culture supernatants were collected using the recorded cell number, volume and incubation time conditions. CTSD, SPP1 and TNF-α were quantified with human ELISAs in duplicate and four-parameter logistic standard curves. In the tumor-conditioned medium exposure experiment, analytes were measured in the medium recovered after exposing macrophage-like cells to CRC cell-conditioned medium. These concentrations are therefore reported as total extracellular CTSD, SPP1 and TNF-α in the recovered medium, without attributing them exclusively to macrophage secretion; input tumor cell-conditioned medium and cell-free controls were not assayed. In the subsequent CTSD knockdown experiment, cells were washed before collecting the medium under fresh culture conditions, as described below.

### 2.13. Macrophage-Conditioned Medium Treatment of CRC Cells

Following HT-29-CM exposure and siRNA treatment, macrophage-like cells were washed to remove the previous treatment medium and incubated in fresh collection medium for 24 h. The cleared supernatants from si-NC and si-CTSD cells were collected under corresponding culture and collection conditions and then applied to CRC recipient cells. This design compares the tumor-supporting activities of newly collected media from otherwise similarly conditioned macrophage-like cells differing in CTSD silencing, thereby limiting direct transfer of the original HT-29-conditioned medium. Any observed recipient cell phenotype may reflect CTSD-associated remodeling of the macrophage-conditioned medium rather than a direct action of extracellular CTSD alone.

### 2.14. CCK-8 Assay

CRC cells were seeded in 96-well plates and treated with TAM-siNC-CM or TAM-siCTSD-CM. Cell viability was measured at 24, 48 and 72 h using CCK-8 reagent, with separate wells for each time point. Absorbance was measured at 450 nm after subtraction of the mean blank well signal.

### 2.15. Transwell Invasion Assay

CRC cell invasion was measured using Matrigel-coated Transwell inserts. After incubation, non-invaded cells were removed and invaded cells were fixed, stained with crystal violet and imaged under identical acquisition settings. Multiple fields from each insert were counted and averaged to obtain one value per experiment.

### 2.16. Colony Formation Assay

CRC cells were seeded at low density and maintained in TAM-siNC-CM or TAM-siCTSD-CM until visible colonies formed. Colonies were fixed, stained with crystal violet and counted using the same threshold in both groups. Colony numbers were normalized to the TAM-siNC-CM group.

### 2.17. Statistical Analysis

All computational analyses were performed in R unless stated otherwise. Gene-level single-cell *p* values were adjusted by the Benjamini–Hochberg procedure. Cell-level Wilcoxon results should be interpreted cautiously because cells within individuals are not independent biological replicates. Kaplan–Meier comparisons used nominal two-sided log rank tests; Cox effect sizes are reported as hazard ratios (HRs) with 95% confidence intervals. Apparent gene-level C-indices were supplemented by 100 repeats of event-stratified ten-fold cross-validation of prespecified Cox models; no external validation was performed. Overall survival was defined as time from diagnosis to death from any cause, censored at last follow-up; TCGA survival annotation included 599 records (122 deaths), the gene expression survival subset 591 cases (121 deaths; 79.5% censored) and the Mac_Scissor+ score survival subset 517 cases. Among censored records, the median reported follow-up was 731 days; median observed time across the clinical annotation was 670 days and maximum follow-up was 4502 days. The 517-person score dataset and selected cutoff were reused for a median cutoff sensitivity comparison. For the experimental assays, qPCR DeltaCt, blot densitometry, ELISA, invasion, colony formation and CCK-8 data were analyzed as originally specified (one-way ANOVA/Dunnett, Welch *t*-test or two-way ANOVA/Sidak as appropriate). Results are mean +/− SEM; *p* < 0.05 was treated as a nominal threshold. Patient-level pseudobulk expression testing and repeated internal Cox cross-validation were added; no independent cohort, prospective validation or nested Scissor feature selection validation was performed.

## 3. Results

### 3.1. Single-Cell Profiling Defined the Colorectal Cancer Cellular Landscape

The single-cell dataset contained 10 normal and 23 tumor colorectal tissue samples; 45,520 cells passed quality control. Quality control and integration plots are shown in Figure S1. A sample-level analysis (Figure S2) showed a median macrophage fraction of 6.55% in tumors (range 0.96–19.49%) and 2.00% in normal samples (1.29–3.12%; Wilcoxon *p* = 0.00030). Among 10 matched tumor–normal patients, nine showed a higher macrophage proportion in tumor tissue (paired Wilcoxon *p* = 0.0039). The cross-sectional variability and paired outlier preclude claiming universal macrophage enrichment.

**Figure 1:**
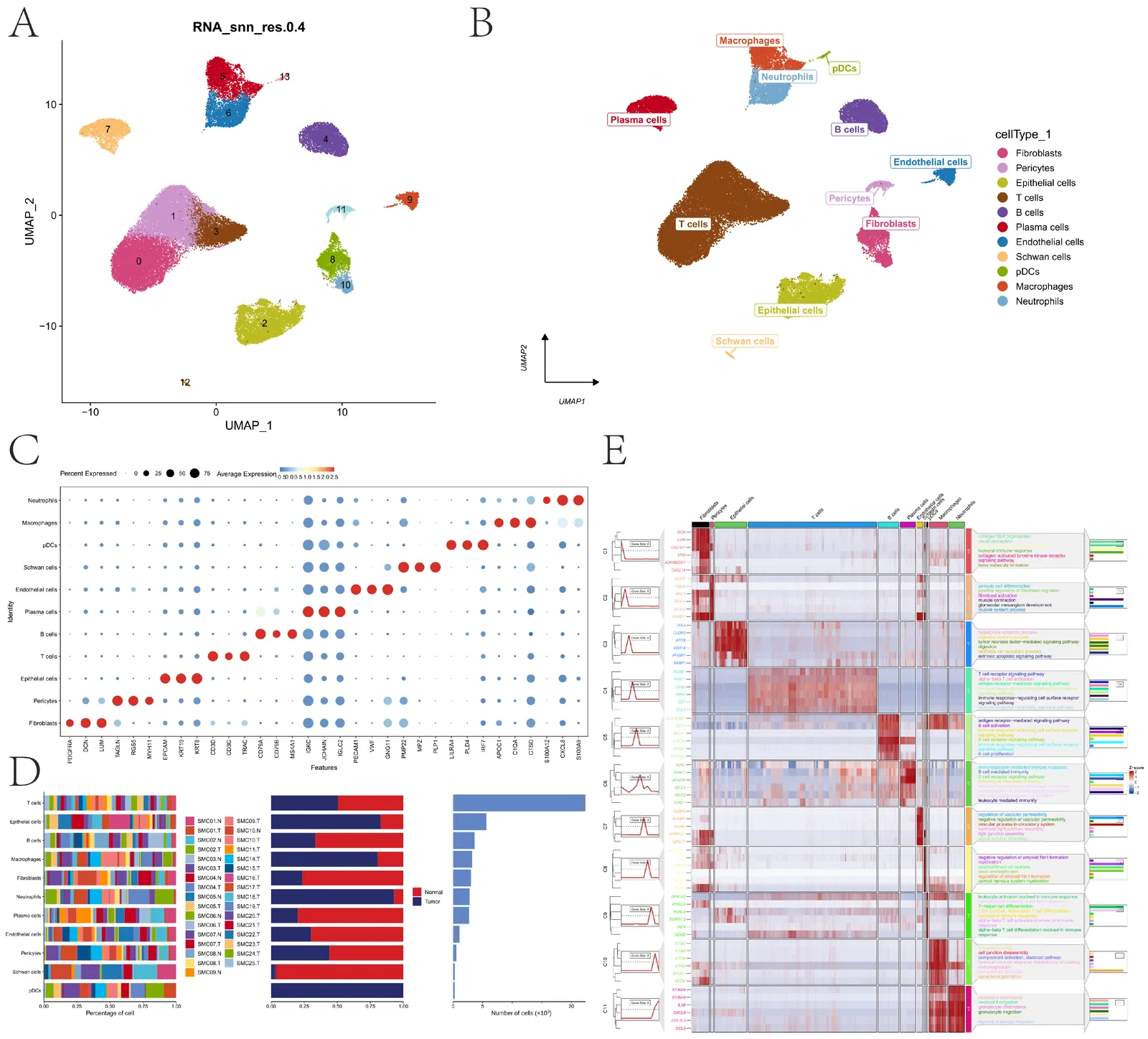
Single-cell quality control, clustering, annotation and cell type functional enrichment. (**A**) UMAP visualization of the integrated single-cell dataset after quality control and Harmony correction. (**B**) Annotation of major cell classes based on canonical markers and reference-supported cell identities. (**C**) Dot plot showing representative marker gene expression across annotated cell classes. (**D**) Cell type proportion summary across colorectal tissue samples and disease groups. (**E**) Functional enrichment of marker genes from major cell classes.

Unsupervised clustering yielded 14 clusters, annotated as 11 major cell classes: fibroblasts, pericytes, epithelial cells, T cells, B cells, plasma cells, endothelial cells, Schwann cells, plasmacytoid dendritic cells, macrophages and neutrophils (Figure 1). The expanded figures show 12 categories when macrophages are displayed separately as Mac_Scissor+ and other macrophages. Macrophages were distinguished from neutrophils and pDCs by the combination of C1QA/C1QB, APOC1 and CSF1R with complementary lineage markers; neither CTSD nor CD68 alone was considered specific (Figures S3 and S4). Malignant and nonmalignant epithelial cells were not separated, so epithelial-specific cell communication cannot be assumed to represent signaling to malignant CRC cells. Among the 23 tumor single-cell specimens with available MSI classification, there were four MSI-H and 19 MSS samples. In this small exploratory sample-level subset, macrophage fraction and Mac_Scissor+ share tended to be greater in MSI-H tumors, but differences did not reach *p* < 0.05 (Figure S5).

Marker genes from each cell population were identified with FindAllMarkers and analyzed for functional enrichment (Figure 1E). Macrophage markers were enriched in complement activation, humoral immune response, tissue remodeling and cell junction-related processes. Phenotype-associated screening of the major cell compartments prioritized macrophages for downstream analysis based on the survival separation of the Scissor-defined macrophage partition. In the supplied exploratory screening of other compartments, Fib_Scissor+ and End_Scissor+ showed nominal optimized cutoff survival *p* values of 0.062 and 0.066, respectively; no complete cell class-wide survival screening summary or adjustment for multiple screened compartments was supplied.

### 3.2. Scissor Identified an Outcome-Associated Macrophage State with Cutoff-Sensitive Survival Separation

**Figure 2:**
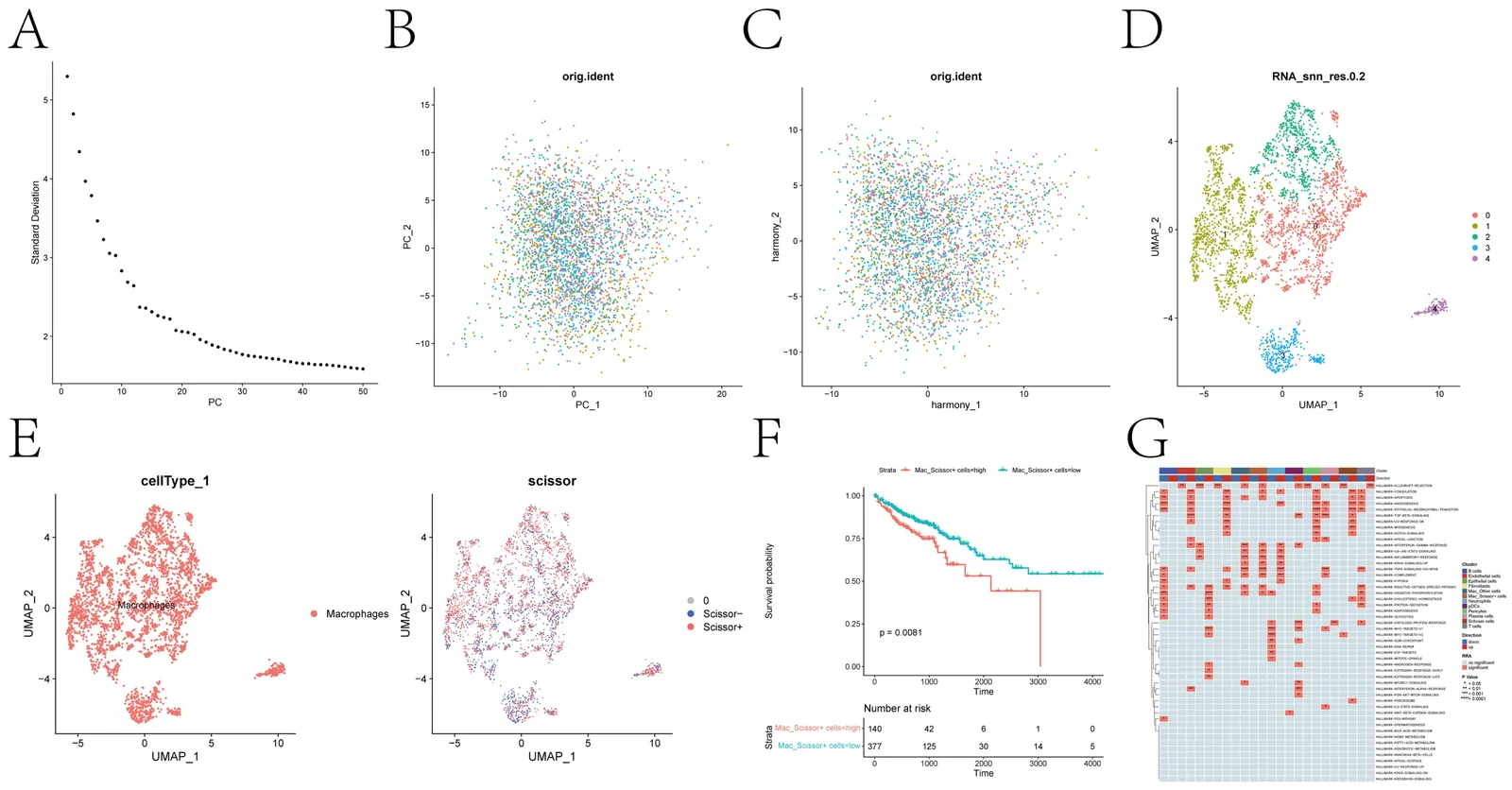
Macrophage reclustering, Scissor outcome-associated mapping, within-cohort survival score comparison and irGSEA activity. (**A**–**D**) Macrophage dimensionality reduction, batch correction, clustering and visualization. (**E**) Scissor+ and Scissor− macrophages and unselected background cells, defined using TCGA overall survival outcomes. (**F**) Exploratory Kaplan–Meier comparison of TCGA cases stratified by a Mac_Scissor+ ssGSEA transcriptional program score at an optimized cutoff; the plot does not validate prognostic performance and the fixed-median sensitivity result appears in Figure S6. (**G**) irGSEA pathway activity patterns. The score is not a measured cell abundance fraction.

Macrophages from all 33 specimens were reclustered into five transcriptional subclusters (Figure 2A–D). Scissor labeled 1912 of 3195 macrophages as Mac_Scissor+ (59.8%), 1271 as Mac_Scissor− (39.8%) and 12 as background/unselected (0.4%) (Figure 2E). Of the Scissor+ macrophages, 1832/1912 (95.8%) originated from tumor tissue; corresponding tumor and normal counts were 1832/80 (Scissor+), 1067/204 (Scissor−) and 12/0 (background). These survival-associated labels are distinct from the five unsupervised macrophage clusters and were obtained using the TCGA overall survival phenotype, which creates outcome-informed selection.

The Mac_Scissor+ ssGSEA signature score in 517 TCGA cases showed a nominal association with overall survival when dichotomized at the optimized cutoff of 0.0382 (high *n* = 140, low *n* = 377; log-rank *p* = 0.0081; univariable HR = 1.72, 95% CI 1.15–2.60; Figure 2F and Figure S6A). In a sensitivity analysis using a fixed median score cutoff of 0.0289, the corresponding groups (258 vs. 259) did not differ significantly (log-rank *p* = 0.537; HR = 0.88, 95% CI 0.60–1.31; Figure S6B). Therefore, the survival separation is cutoff-dependent; further, the cell state signature and optimal cutoff had been constructed with reference to TCGA survival and the *p* values do not represent out-of-sample validation. Exploratory similarly optimized scores for Fib_Scissor+ and End_Scissor+ returned nominal *p* = 0.062 and *p* = 0.066, respectively, in the supplied analysis; these exploratory comparisons were not adjusted for screening multiple cell compartments. Hallmark irGSEA patterns showed shared as well as state-specific pathways; similar or nonsignificant pathway signals do not negate differential expression of individual genes. Available TCGA MSI/mutation subgroup summaries are shown in Figure S7. The complementary KEGG single-cell analysis is shown in Figure S8.

irGSEA showed distinct pathway activity patterns across macrophage-associated states (Figure 2G). The top differentially enriched gene sets were concordant across the rank-based scoring methods included in the robust rank aggregation.

### 3.3. Mac_Scissor+ Macrophages Showed Distinct Communication and Differentiation Profiles

**Figure 3:**
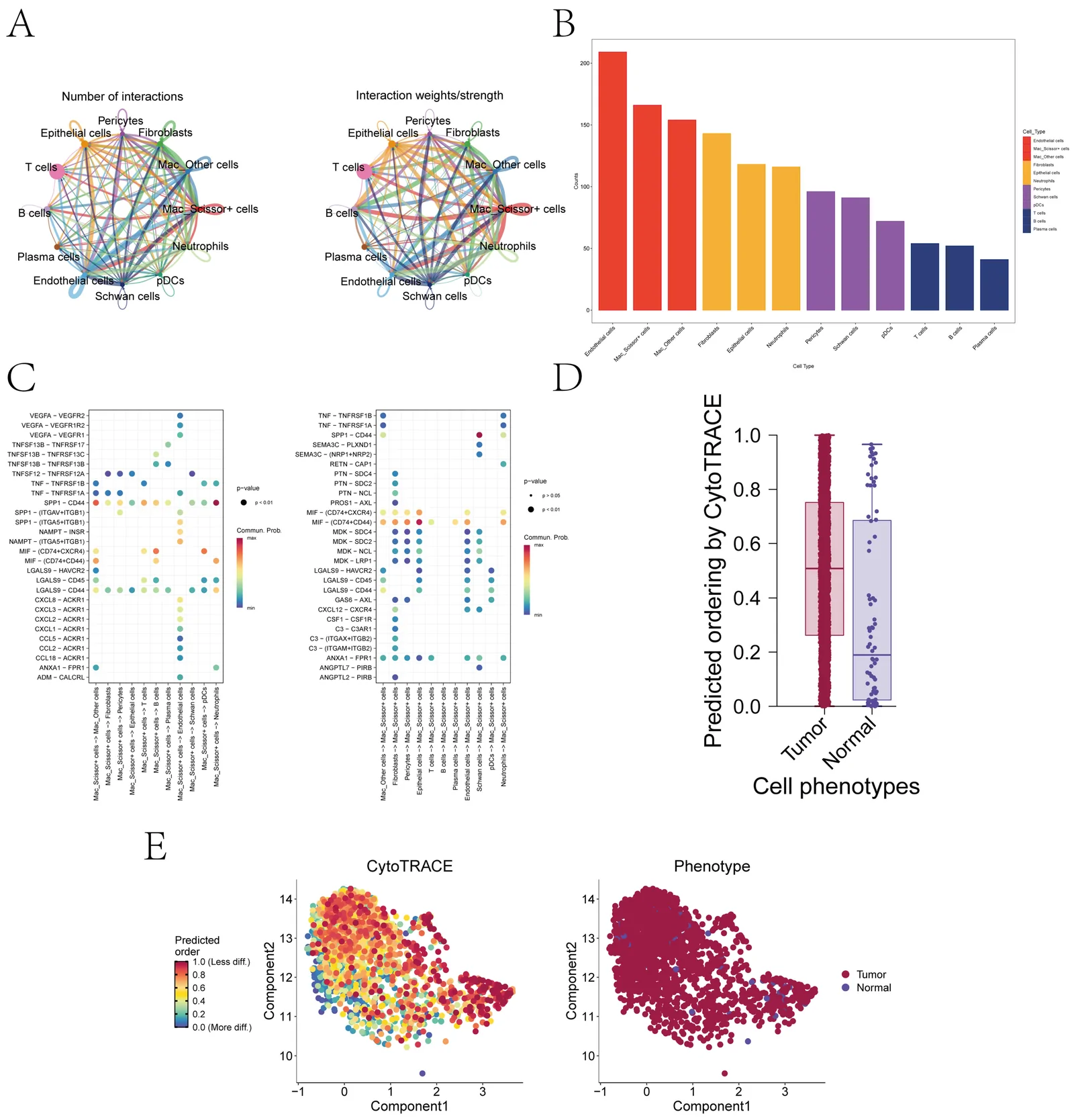
Communication, CytoTRACE and differentiation state analyses of Mac_Scissor+ macrophages. (**A**,**B**) CellChat network summaries showing intercellular communication among annotated cell classes. (**C**) Representative ligand–receptor axes involving Mac_Scissor+ macrophages as signaling source or target cells. (**D**,**E**) CytoTRACE-based differentiation potential and differentiation state visualizations within the Mac_Scissor+ macrophage compartment.

CellChat suggested extensive intercellular ligand-receptor networks (Figure 3A,B). Endothelial cells had the highest aggregate predicted communication score and the Mac_Scissor+ macrophage category ranked next in this descriptive comparison. This analysis does not identify a dominant causal sender or a specific malignant epithelial target. Several inferred SPP1-integrin/CD44 and LGALS9-CD44 interactions connected Mac_Scissor+ cells with the broader epithelial category (Figure 3C), but tumor cell identity within that category was not independently resolved.

Ligand–receptor summaries identified SPP1-integrin/CD44, LGALS9-CD44/CD45/HAVCR2, MIF-CD74-CD44/CD74-CXCR4, TNF-TNFRSF1A/TNFRSF1B and VEGFA-VEGFR1/VEGFR2 interactions involving Mac_Scissor+ macrophages as signaling source or target cells (Figure 3C). These interactions span matrix adhesion, immune regulation, inflammatory signaling and vascular communication.

Within the two displayed tissue origin groups, tumor-derived Mac_Scissor+ macrophages showed higher CytoTRACE-predicted differentiation-associated potential than normal-derived Mac_Scissor+ macrophages (Figure 3D,E). This cross-sectional comparison neither establishes stemness nor reconstructs actual macrophage lineage transitions.

### 3.4. Pseudotime Analysis Revealed Transcriptional Heterogeneity Within Mac_Scissor+ Macrophages

**Figure 4:**
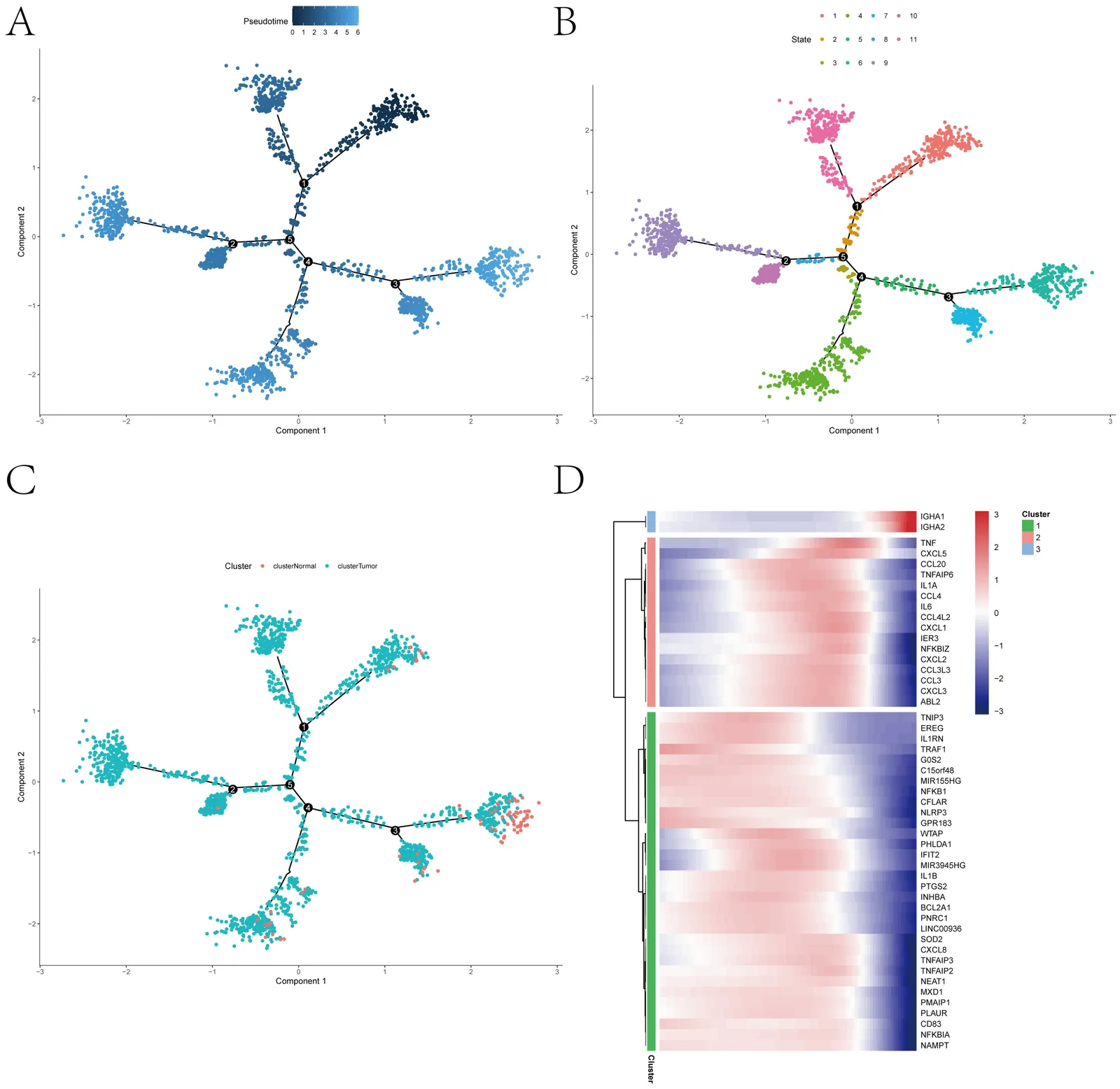
Pseudotime trajectory and pseudotime-associated gene expression programs in Mac_Scissor+ macrophages. (**A**) Monocle trajectory colored by pseudotime. (**B**) Branch state distribution across the inferred trajectory. (**C**) Group-level distribution of macrophage states across pseudotime space. (**D**) Heatmap of genes varying along pseudotime, grouped into major expression clusters. Pseudotime indicates an inferred transcriptional ordering and is not interpreted as direct lineage tracing.

Monocle ordered Mac_Scissor+ macrophages along a branched transcriptional trajectory (Figure 4A–C). The branches represent transcriptionally distinct states within the Mac_Scissor+ compartment; the cross-sectional design does not establish developmental lineage relationships.

Genes varying along the inferred ordering formed three expression clusters (Figure 4D). TNIP3 was more prominent toward one end of the trajectory, whereas IGHA1 increased in later pseudotime regions. These patterns document structured transcriptional heterogeneity within Mac_Scissor+ macrophages.

### 3.5. Spatial Transcriptomics Described the Tissue Context of the Macrophage Program

Spatial profiling of one primary tumor and two adjacent normal sections from GSE280313 identified 12 spatial clusters after preprocessing (Figure S9). The sections corresponded to the deposited tumor and normal tissue samples, and their unequal composition (one tumor, two normals) limits between-condition comparison.

**Figure 5:**
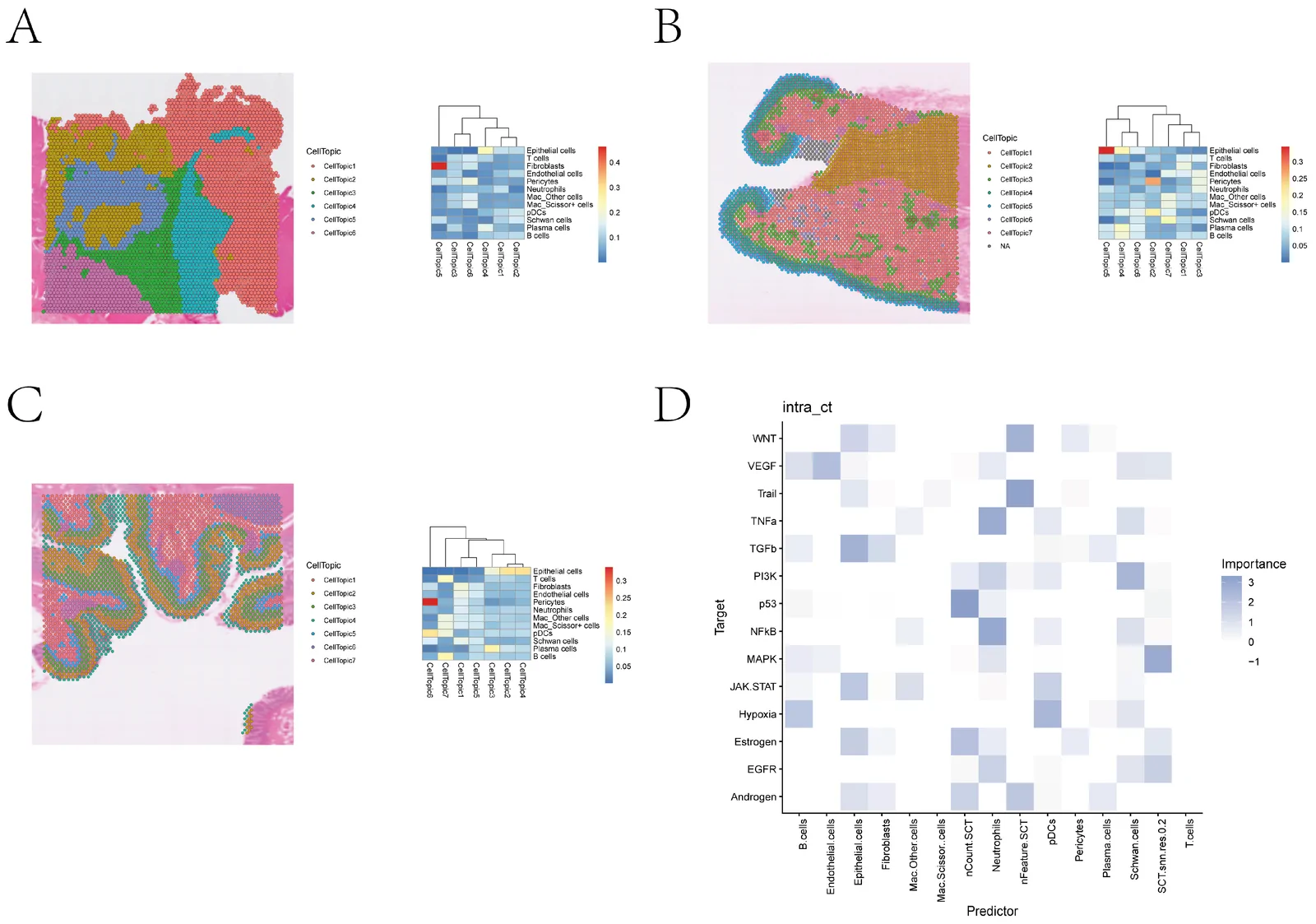
Exploratory SpaTopic domains and corrected PROGENy pathway activity analysis. (**A**) Tumor section, GSE280313, showing SpaTopic spatial domains and descriptive cell composition; (**B**,**C**) two normal-adjacent sections from the same GEO series. (**D**) Corrected PROGENy pathway footprint heatmap with cell population predictors only; technical covariates nCount_SCT, nFeature_SCT and SCT.snn.res.0.2 have been removed from the *x*-axis. These analyses are descriptive: no formal Mac_Scissor+-specific enrichment test against a CellTopic or pathway was performed.

SpaTopic partitioned the three sections into CellTopic-defined spatial domains (Figure 5A–C). The accompanying heatmaps show the estimated representation of major cell populations across topics. Topic composition differed among sections, reflecting spatial heterogeneity across the analyzed tissues.

PROGENy showed variation in pathway activity across spatial and cell type contexts (Figure 5D). Because no formal association test linked Mac_Scissor+ abundance with individual CellTopics or PROGENy pathways, these analyses were restricted to tissue-context description.

### 3.6. Random Survival Forest Analysis Prioritized CTSD, HSPA1A and SPP1

At single-cell resolution, differential expression analysis between 1912 Mac_Scissor+ and 1271 Mac_Scissor− macrophages identified 49 genes at avg_log2FC > 0.25 and adjusted *p* < 0.05. SPP1 (avg_log2FC = 1.34; 59.0% versus 38.2% positive cells; adjusted *p* = 3.39 × 10^−31^) had the largest shift among the three prioritized genes. CTSD (0.36; 92.5% versus 86.5%; adjusted *p* = 1.54 × 10^−12^) and HSPA1A (0.75; 64.1% versus 54.3%; adjusted *p* = 2.81 × 10^−12^) showed relatively modest per-cell shifts despite small cell-level *p* values (Figures S10–S12). The small cell-level effect sizes and within-donor correlations motivated independent-unit pseudobulk comparisons, reported separately below; tumor-versus-normal expression differences do not validate the survival-selected Mac_Scissor+ versus Mac_Scissor− contrast. The distribution of SPP1 expression across annotated cell populations is shown in Figure S13.

**Figure 6:**
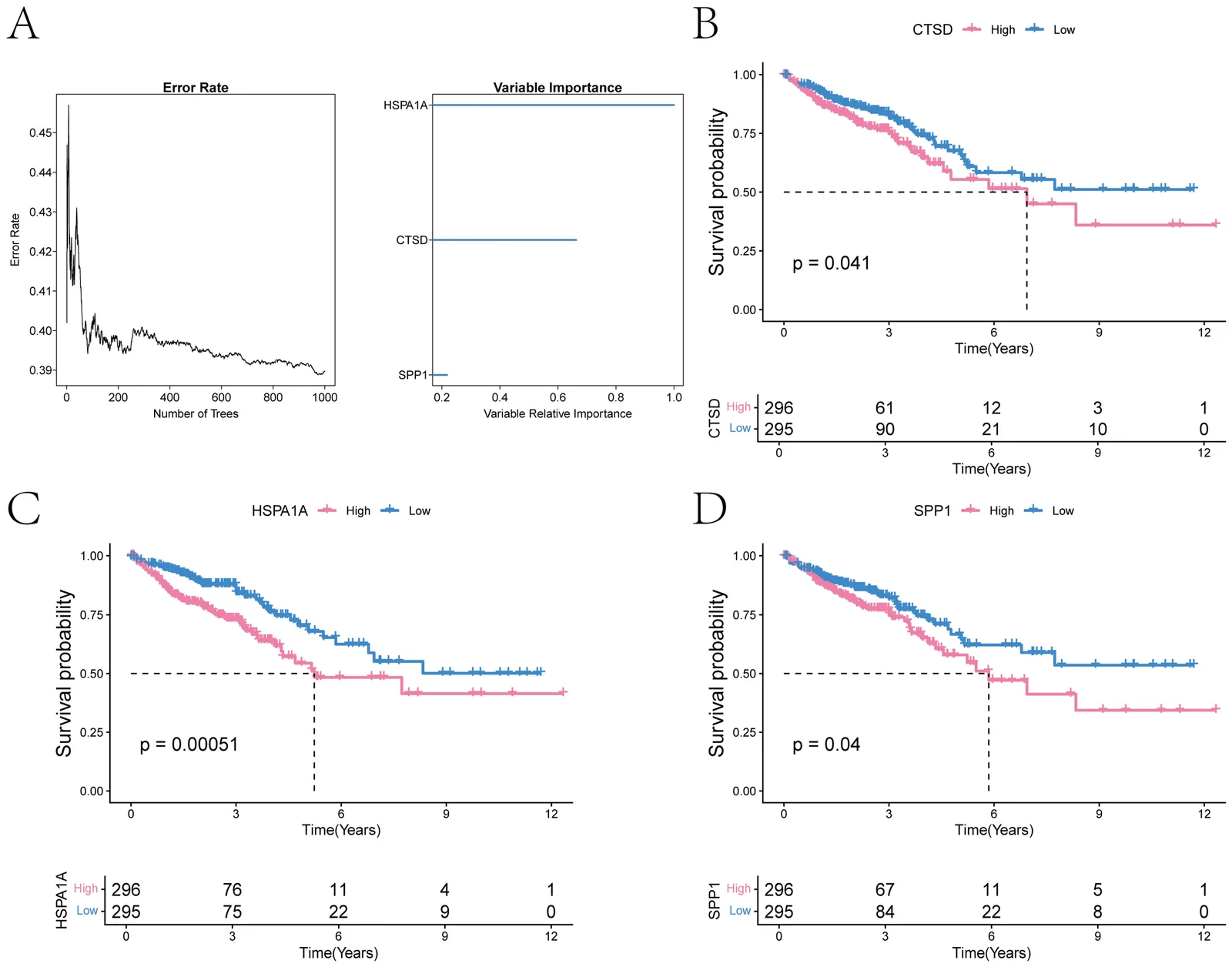
Exploratory random survival forest feature prioritization and gene-level survival associations. (**A**) Out-of-bag error over 1000 trees and relative variable importance for 49 single-cell-derived candidate genes (top VIMP normalized to 1; display threshold > 0.20). (**B**–**D**) Nominal univariable Kaplan–Meier results for CTSD, HSPA1A and SPP1 in the 591-person TCGA subset (high 296, low 295, with 121 deaths). Per-gene univariable and clinicopathologically adjusted Cox effect estimates are provided in Supplementary Table S1 and Figure S14. All survival results were evaluated in the discovery data and are not independent validation.

On the matching TCGA bulk expression features for these 49 candidates, random survival forest analysis ranked HSPA1A (relative importance 1.000), CTSD (0.663) and SPP1 (0.218) above the operational threshold of >0.20 (Figure 6A). The out-of-bag error curve approached a plateau as tree count increased; however, OOB prediction error does not supply nested validation of outcome-informed gene selection. The gene-level median-split Kaplan–Meier tests (*n* = 591; 121 deaths) showed nominal survival associations for CTSD (*p* = 0.041), HSPA1A (*p* = 0.00051) and SPP1 (*p* = 0.040) (Figure 6B–D). These effect estimates may be optimistic given reuse of the TCGA survival endpoint; repeated internal cross-validation results for fixed candidate Cox models are reported below but cannot remove the selection-stage outcome leakage.

The three genes represent distinct features of the macrophage program: SPP1 encodes the matrix-associated secreted protein osteopontin [36], CTSD encodes the lysosomal protease cathepsin D, and HSPA1A encodes a stress-inducible heat shock protein. Their combined exploratory prioritization captured matrix interaction, lysosomal function and stress response components of the Mac_Scissor+ state, but the added tissue-level expression evidence did not support all three candidates equally.

#### 3.6.1. Patient-Level Pseudobulk Expression Supports CTSD and SPP1, but Not HSPA1A

Aggregation of macrophage raw counts by tissue specimen yielded 33 pseudobulk profiles (23 tumor and 10 adjacent normal) from 23 patients; ten patients had paired specimens. Median log2(CPM + 1) expression across tumor versus normal specimens was 11.49 versus 9.84 for CTSD, 9.23 versus 8.17 for HSPA1A, and 11.20 versus 0 for SPP1 (Figure S17; Table S4). In matched pairs, CTSD and SPP1 expression was higher in all 10 tumors (both exact paired Wilcoxon *p* = 0.00195); HSPA1A increased in seven of 10 pairs but was not significant (*p* = 0.131). Paired DESeq2 estimates corroborated CTSD (log2 fold change 1.92; adjusted *p* = 6.14 × 10^−5^) and SPP1 (9.15; adjusted *p* = 5.85 × 10^−21^), whereas HSPA1A remained nonsignificant (1.19; adjusted *p* = 0.246). The normal-adjacent SPP1 median was zero CPM, so no finite ratio of group medians is reported. These specimen-level results establish tumor–normal macrophage expression differences for CTSD and SPP1, not patient-level survival prediction or a direct validation of the Scissor-defined cell state contrast.

#### 3.6.2. Repeated Cox Cross-Validation Reveals Modest Discrimination and Limited Incremental Value

Across 100 repetitions of event-stratified ten-fold cross-validation in 571 complete TCGA cases (113 deaths), the mean held-out C-indices were 0.550 for CTSD, 0.612 for HSPA1A and 0.533 for SPP1 when modeled individually per SD of expression; the three-gene model reached 0.604 (Figure S18; Table S5). The corresponding full-data apparent C-indices were 0.556, 0.616, 0.542 and 0.619, respectively. The age/sex/stage reference model had a cross-validated C-index of 0.714, versus 0.717 (CTSD), 0.723 (HSPA1A), 0.713 (SPP1) and 0.722 (all three genes) for clinical covariate-adjusted models. Thus, adding all three genes changed mean held-out concordance by only +0.008 compared with the clinical-only reference. Median split single-gene models performed less well (0.526, 0.576 and 0.514 for CTSD, HSPA1A and SPP1, respectively). Between-partition SDs of pooled C-indices ranged from 0.0025 to 0.0083; this quantifies variability from re-partitioning, not population generalizability. Because gene selection had already used the TCGA survival phenotype, the cross-validation addresses fixed-model instability and apparent-to-held-out optimism, but not leakage-free validation of biomarker discovery.

### 3.7. CTSD, HSPA1A and SPP1 Correlated with Distinct Immune Cell Profiles

**Figure 7:**
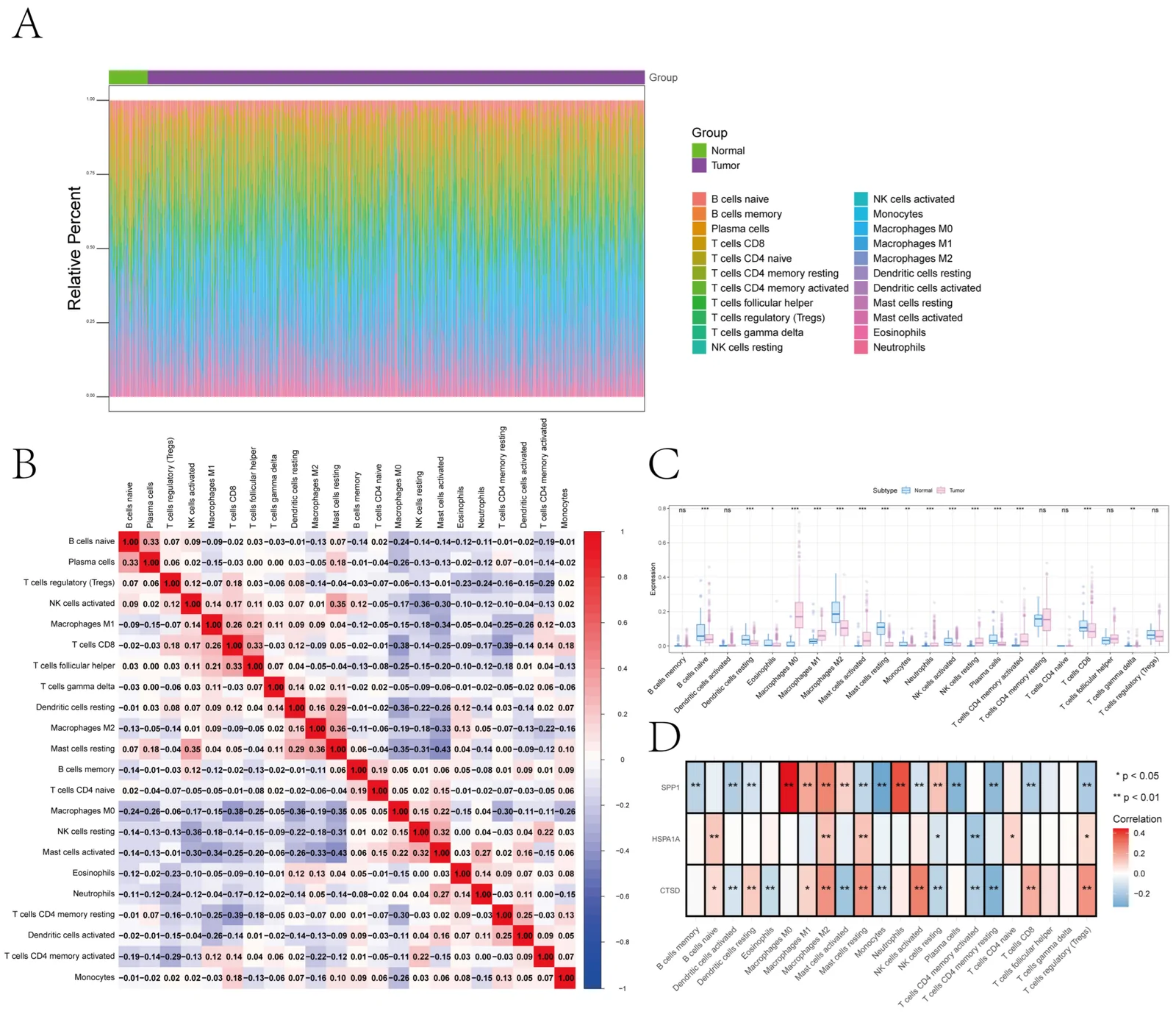
Bulk immune deconvolution and correlations with candidate genes. (**A**) Relative LM22 immune cell estimates; (**B**) correlations among estimated relative fractions, which are affected by compositional constraints and related reference profiles; (**C**) descriptive tumor-versus-normal fraction comparisons; (**D**) associations between CTSD, HSPA1A or SPP1 and inferred fractions. LM22 M0/M1/M2 reference phenotypes must not be equated with Scissor-selected single-cell states. * Indicates *p* < 0.05 ** Indicates *p* < 0.01 *** Indicates *p* < 0.001.

CIBERSORT estimated relative frequencies of the 22 LM22 reference immune phenotypes (Figure 7). Compared with controls, tumors showed relatively higher inferred M0 macrophages and activated mast cells and lower inferred naive B-cell and M2 macrophage fractions. The LM22 M2 category is not equivalent to SPP1+ or Mac_Scissor+ macrophages and cannot be interpreted as a universal protumor cell state. Because inferred fractions sum to one and reference cell profiles can overlap, correlations between the inferred cell categories are compositional and do not independently validate or invalidate single-cell lineage annotations.

CTSD, HSPA1A and SPP1 showed distinct correlations with inferred immune cell fractions (Figure 7D). CTSD correlated positively with naive B cells, CD8 T cells, regulatory T cells, activated NK cells, Macrophages M1, Macrophages M2, resting dendritic cells and resting mast cells, and negatively with resting and activated CD4 memory T cells, resting NK cells, monocytes, activated dendritic cells, activated mast cells and eosinophils. HSPA1A correlated positively with naive B cells, naive CD4 T cells, regulatory T cells, Macrophages M2 and resting mast cells, and negatively with activated CD4 memory T cells and resting NK cells. SPP1 correlated positively with resting NK cells, Macrophages M0, Macrophages M1, Macrophages M2, activated mast cells and neutrophils, and negatively with memory B cells, plasma cells, CD8 T cells, resting CD4 memory T cells, regulatory T cells, activated NK cells, monocytes, resting and activated dendritic cells and resting mast cells.

CTSD, HSPA1A and SPP1 showed different bulk immune infiltration profiles rather than a uniform macrophage-associated pattern. The correlation analysis did not determine the direction of immune cell recruitment or regulation.

### 3.8. CTSD, HSPA1A and SPP1 Were Associated with Distinct Pathway Profiles

**Figure 8:**
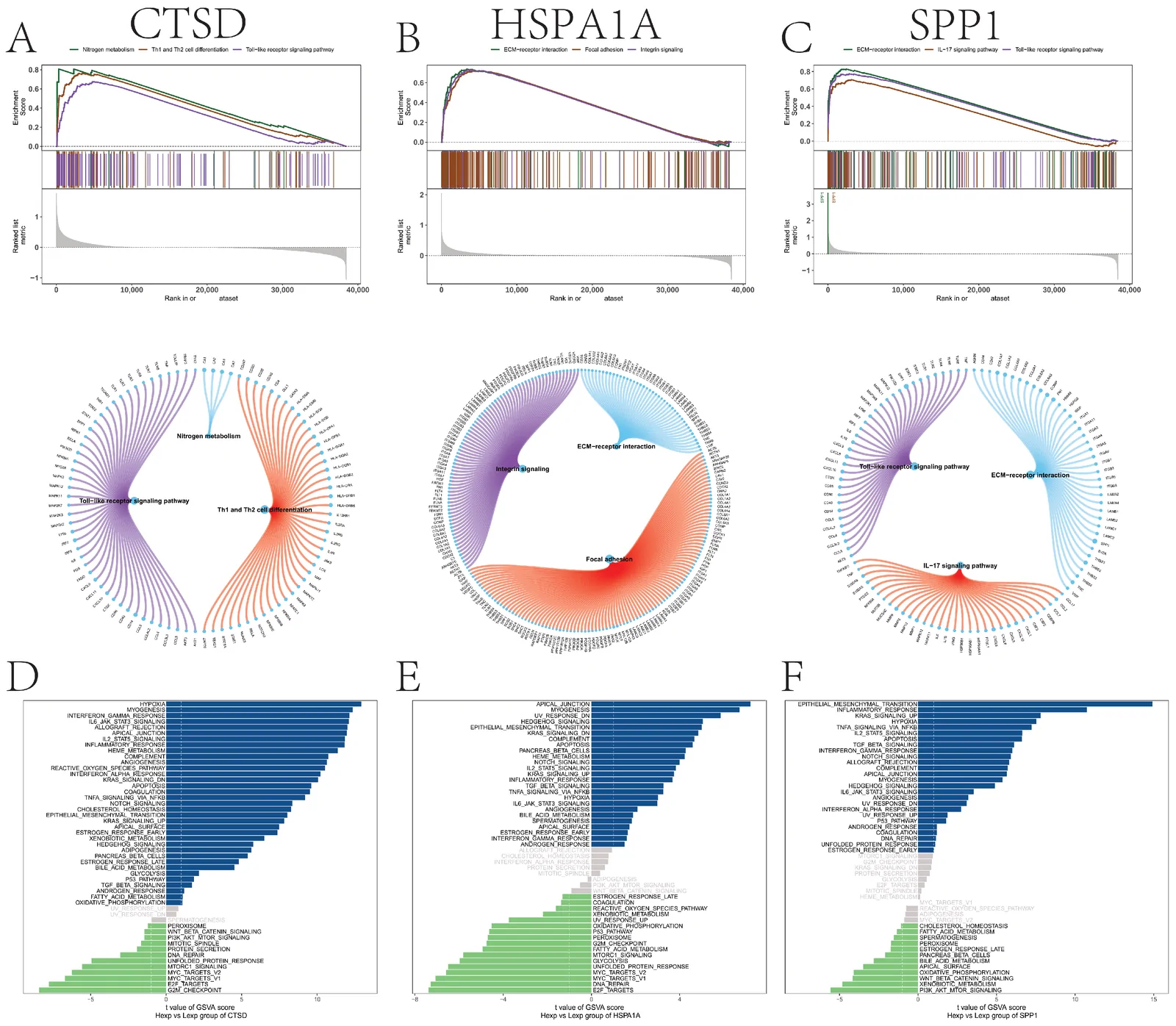
GSEA and GSVA pathway analyses for CTSD, HSPA1A and SPP1. (**A**–**C**) GSEA results for CTSD-, HSPA1A- and SPP1-stratified tumor samples. (**D**–**F**) GSVA pathway activity patterns associated with CTSD, HSPA1A and SPP1 expression. Enrichment analyses were performed using predefined gene sets.

GSEA and GSVA characterized different levels of association with candidate gene expression. For Figure 8A–C, bulk tumors were divided at the median expression of each candidate gene; all other genes were ranked by group comparison and assessed against human KEGG pathway sets with clusterProfiler::gseKEGG (organism = ‘hsa’) [31]. For Figure 8D–F, sample-level GSVA scores were obtained using the MSigDB Hallmark collection and high- versus low-expression groups were compared using limma. These observational pathway results are not perturbation-based pathway validation.

CTSD-associated GSEA included Th1 and Th2 cell differentiation, Toll-like receptor signaling, nitrogen metabolism, phagosome, complement and coagulation cascades, hematopoietic cell lineage, Th17 differentiation, cytokine–cytokine receptor interaction and antigen-related pathways (Figure 8A). GSVA associated CTSD expression with hypoxia and myogenesis programs (Figure 8D).

HSPA1A-associated GSEA included focal adhesion, integrin signaling, extracellular matrix receptor interaction, cytoskeletal regulation and actin cytoskeleton pathways, while ribosome, cell cycle and oxidative phosphorylation were negatively enriched (Figure 8B). GSVA associated HSPA1A expression with apical junction and myogenesis programs (Figure 8E).

SPP1-associated GSEA included extracellular matrix receptor interaction, Toll-like receptor signaling, IL-17 signaling, phagosome, integrin signaling, focal adhesion, cytokine–cytokine receptor interaction and antigen processing and presentation (Figure 8C). GSVA associated SPP1 expression with epithelial–mesenchymal transition and inflammatory response (Figure 8F).

### 3.9. CRC Cell-Conditioned Media Altered CTSD, SPP1 and HSPA1A in Macrophage-like Cells

**Figure 9:**
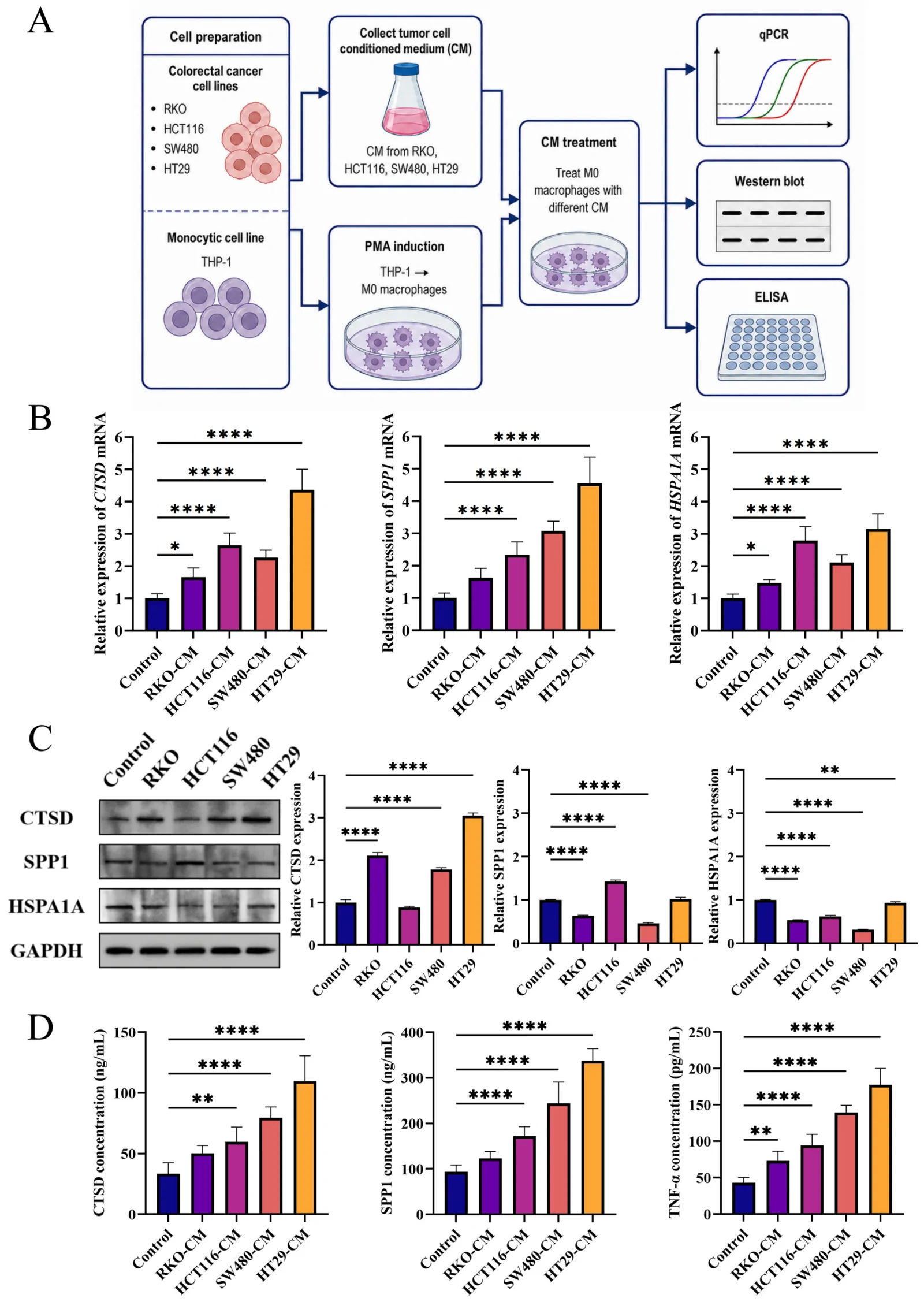
Responses of PMA-differentiated THP-1-derived macrophage-like cells to colorectal cancer cell-conditioned media. (**A**) Workflow, including the differentiated macrophage-like cell group maintained in unconditioned control medium. (**B**) CTSD, SPP1 and HSPA1A mRNA expression. (**C**) Representative bands and GAPDH-normalized quantification of CTSD, SPP1 and HSPA1A in THP-1-derived macrophage-like cell lysates. Western blot quantification represents three original biological experiments per condition; the full numerical band readings and representative source exposures are supplied in Table S2 and Figure S15, respectively. (**D**) Total CTSD, SPP1 and TNF-α measured in culture supernatants (not attributed exclusively to macrophage secretion because input tumor cell-conditioned medium and cell-free controls were not assayed). qPCR and ELISA, *n* = 8 per group as recorded; Western blot, *n* = 3 independent biological experiments. Data are mean ± SEM; one-way ANOVA followed by Dunnett-adjusted comparisons versus control. * *p* < 0.05, ** *p* < 0.01, **** *p* < 0.0001.

PMA-differentiated THP-1-derived macrophage-like cells were exposed to conditioned media from RKO, HCT116, SW480 and HT-29 cells, with PMA-differentiated cells maintained without CRC-conditioned medium serving as the baseline control (Figure 9A). This design directly compared how soluble signals from four CRC cell lines affected a common macrophage-like cell model, without assigning the baseline or tumor-conditioned cells to canonical M1/M2 categories.

Western blot densitometry was verified against the three original biological replicate readings (Figure 9C and Table S2). Relative to the unconditioned macrophage-like cell control (1.00), CTSD/GAPDH was 2.11 in RKO-CM, 0.89 in HCT116-CM, 1.78 in SW480-CM and 3.05 in HT-29-CM. The respective SPP1/GAPDH values were 0.63, 1.43, 0.46 and 1.03, and HSPA1A/GAPDH values were 0.54, 0.63, 0.32 and 0.93. Thus, HT-29-CM elicited the largest CTSD protein response, whereas SPP1 and HSPA1A displayed different, nonparallel protein-level patterns. The control and all analyzed lysates were derived from PMA-differentiated THP-1 macrophage-like cells; no specifically polarized M1 or M2 identity was established.

ELISA showed higher total extracellular CTSD, SPP1 and TNF-α concentrations after exposure to the CRC cell-conditioned media than in the unconditioned macrophage control, with the strongest combined extracellular signal in the HT-29-CM condition (Figure 9D). These findings show that different tumor-derived conditioned media create distinct soluble factor environments around the macrophage-like cells. Because the input CRC-conditioned media were not measured separately, Figure 9D is interpreted as total recovered analyte rather than macrophage-specific secretion. Together with the pronounced macrophage cell CTSD protein response in Figure 9C, these findings supported selection of the HT-29-CM-conditioned model for CTSD knockdown.

### 3.10. CTSD Knockdown Reduced the Tumor-Supporting Activity of Macrophage-Conditioned Medium

**Figure 10:**
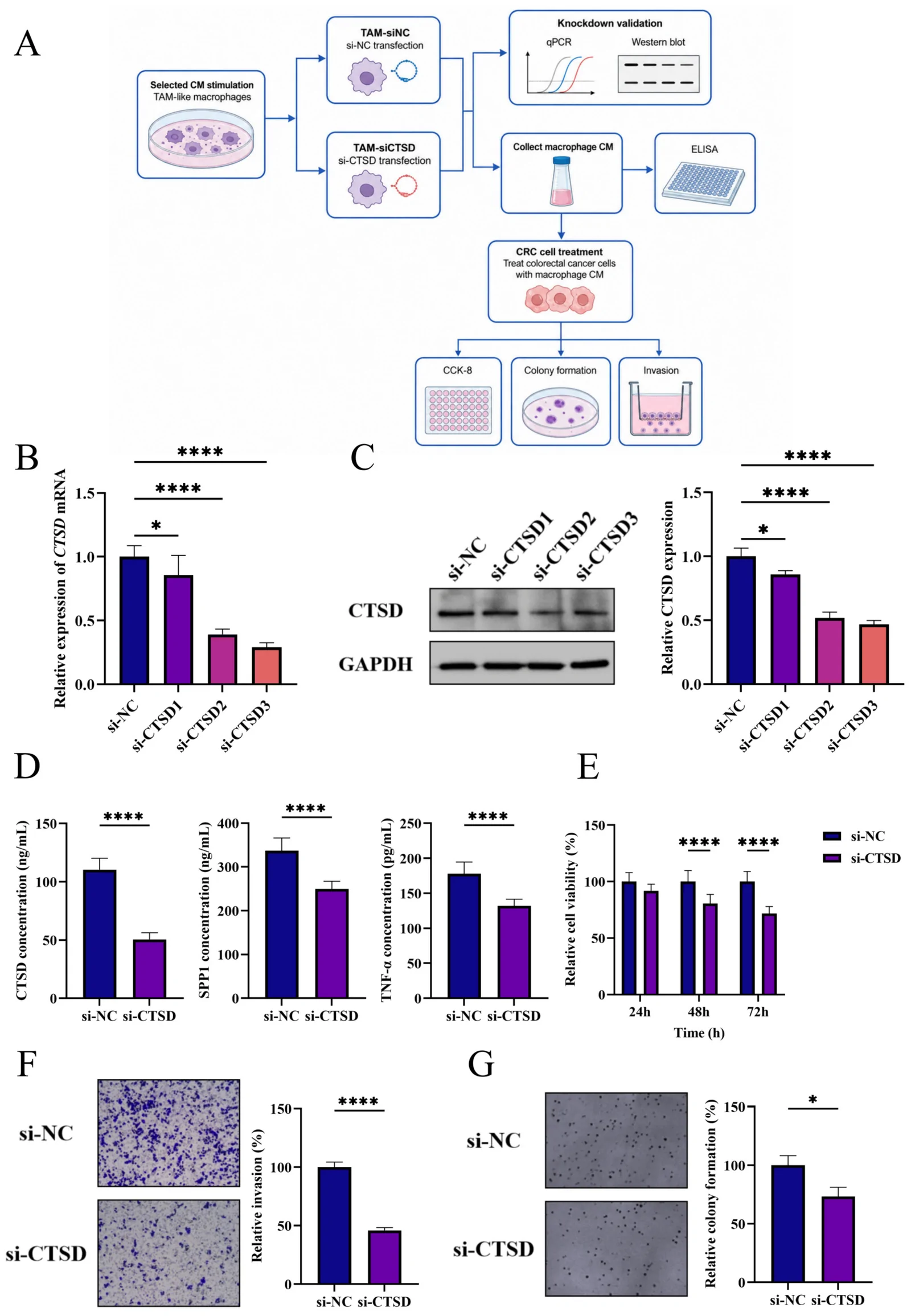
Effects of CTSD knockdown in HT-29-CM-conditioned THP-1-derived macrophage-like cells and their subsequently collected conditioned medium on colorectal cancer cells. (**A**) Workflow, including medium washout and collection after siRNA exposure. (**B**) qPCR measurements for three CTSD-targeting siRNAs. (**C**) Representative CTSD and GAPDH bands with quantitative analysis based on three original biological replicates per group (full intensities and ratios: Table S3; available representative original source exposures: Figure S16). (**D**) CTSD, SPP1 and TNF-α in macrophage-conditioned supernatants. (**E**) Colorectal cancer cell viability at 24, 48 and 72 h. The si-NC versus si-CTSD comparison evaluates the effect of CTSD perturbation on the bioactivity of subsequently collected macrophage-conditioned medium. (**F**) Matrigel Transwell invasion and quantitative analysis; (**G**) colony formation and quantitative analysis after exposure to macrophage-conditioned media. qPCR and ELISA, *n* = 8 per group; Western blotting, invasion and colony formation, *n* = 3 independent experiments; CCK-8, *n* = 8 per group. Data are mean ± SEM. Knockdown screening: one-way ANOVA with Dunnett’s test; ELISA, invasion and colony formation: Welch’s unpaired *t*-test; CCK-8: two-way ANOVA with Sidak-adjusted pairwise comparisons. * *p* < 0.05, **** *p* < 0.0001.

HT-29-CM-exposed THP-1-derived macrophage-like cells were transfected with non-targeting siRNA (si-NC) or one of three CTSD-targeting siRNAs (Figure 10A). The three original Western blot replicate datasets showed GAPDH-normalized CTSD abundances of 1.000 ± 0.037 (si-NC), 0.858 ± 0.017 (si-CTSD1), 0.518 ± 0.026 (si-CTSD2) and 0.468 ± 0.018 (si-CTSD3; mean ± SEM, *n* = 3). These correspond to protein reductions of 14.2%, 48.2% and 53.2%, respectively. The si-CTSD3 oligonucleotide, which produced the largest protein reduction and strongest transcript suppression, was selected for downstream functional experiments (Figure 10B,C; Table S3; Figure S16).

After medium washout and fresh 24 h collection, CTSD silencing reduced CTSD detected in macrophage-conditioned supernatants and was accompanied by lower SPP1 and TNF-α concentrations (Figure 10D). These coordinated changes indicate that CTSD perturbation alters the soluble factor profile of the HT-29-conditioned macrophage-like cell culture, without establishing that SPP1 or TNF-α is a direct downstream substrate or mediator of CTSD.

Freshly collected conditioned media from si-NC- and CTSD-silenced HT-29-conditioned macrophage-like cells were transferred to CRC recipient cells. Compared with the si-NC-derived medium, the CTSD-silenced macrophage medium reduced CRC cell viability at 48 and 72 h, Matrigel Transwell invasion, and colony formation (Figure 10E–G). The concordant effects across three functional readouts demonstrate that silencing CTSD in the tumor-conditioned macrophage-like cells attenuates the tumor-supporting activity of the resulting conditioned medium.

## 4. Discussion

We identified a Mac_Scissor+ macrophage state enriched for CTSD, HSPA1A and SPP1 and examined the functional consequences of macrophage CTSD silencing in a conditioned medium model. Scissor used TCGA overall survival to assign cell states; the subsequent same-cohort enrichment score comparisons, random survival forest prioritization and Cox associations are internally selected and potentially optimistic. The optimized score cutoff yielded separation whereas a fixed median cutoff did not. Patient-level pseudobulk comparison of all macrophages independently supported tumor-associated CTSD and SPP1 expression, but HSPA1A was not significant in matched specimens. Repeated ten-fold cross-validation of fixed Cox specifications reduced reliance on apparent concordance alone; however, gene-only discrimination was modest and adding the three genes to age, sex and stage changed C-index by only 0.008. Because Scissor/RSF feature selection was not repeated within the training folds, the study still supports candidate discovery rather than validation of a prognostic signature.

Macrophage heterogeneity is not adequately represented by binary M1/M2 nomenclature [9,10,11,12]. The integrated single-cell annotation was supported by C1QA/C1QB, APOC1, CSF1R and other lineage-associated markers, but markers overlap and single-cell state labels are not immutable lineages. SPP1/TREM2-enriched states may reflect matrix remodeling and other tumor-related programs; expression-based correlations cannot establish that these cells execute a particular immunosuppressive function. At the sample level, most matched tumors showed greater macrophage representation than paired normal tissues, though MSI-H strata were small and potentially confounded.

Tumor cell-conditioned media produced distinct CTSD, SPP1 and HSPA1A expression responses in THP-1-derived macrophage-like cells. HT-29-CM produced the largest increase in intracellular CTSD protein, whereas the SPP1 and HSPA1A immunoblot profiles were not parallel. The most important functional observation was made after CTSD knockdown: following removal of the original treatment medium and fresh medium collection, the resulting macrophage-conditioned medium was less effective at supporting CRC cell viability, invasion and colony formation. This sequence moves beyond transcriptomic association to demonstrate that macrophage CTSD perturbation changes the tumor-supporting properties of the model’s secretome, without requiring the assumption of an M2-polarized state. The donor-level pseudobulk tumor–normal analysis supports a reproducible increase in CTSD expression in macrophages across matched tissue specimens, yet it does not establish that CTSD distinguishes survival-defined states within patients or that THP-1 cultures reproduce a particular patient macrophage state. The modest single-cell Scissor+ versus Scissor− CTSD effect therefore remains an important interpretative constraint.

Cathepsin D is a lysosomal protease involved in proteostasis, extracellular matrix-related processes and tumor–stroma biology [37]. In our macrophage-focused perturbation system, si-CTSD3 reduced cellular CTSD and the CTSD concentration in the subsequently collected conditioned medium; the same medium showed lower tumor-supporting activity in complementary CRC cell assays. These findings support a functional role for CTSD in the HT-29-conditioned macrophage-like system. However, a change in CTSD expression can affect multiple components of the macrophage secretome, and the present experiments do not distinguish indirect effects from a direct effect of extracellular CTSD on CRC cells.

The reductions in SPP1 and TNF-α concentrations following CTSD knockdown are consistent with a broader alteration of the macrophage-conditioned soluble milieu. They do not imply a demonstrated CTSD–SPP1 signaling hierarchy. The initial supernatant measurements in Figure 9D reflect medium collected after exposure to CRC cell-conditioned medium, and thus include potential tumor cell-derived input; accordingly, our inference about macrophage CTSD activity relies chiefly on Figure 10, in which the original treatment medium was removed before supernatant collection. Direct attribution of the Figure 9D ELISA signal to macrophages would require input medium and cell-free controls that were not available in this study.

HSPA1A is a stress-inducible chaperone whose bulk expression correlated with adhesion, matrix and cytoskeletal pathways [38]. Its role here is associative; paired pseudobulk comparisons did not confirm a significant HSPA1A increase in tumor macrophages, and no HSPA1A perturbation was performed. Although HSPA1A showed the highest candidate gene cross-validated concordance in bulk TCGA, this result does not resolve outcome-informed gene selection bias or demonstrate macrophage-specific expression as the cause of the survival association. Similarly, the SPP1 signals were not specific to macrophages, and the available figures show other cell populations contributing to SPP1 expression (Figure S13). SPP1-expressing macrophages were not categorized as canonical M2 cells on the basis of expression alone.

Spatial deconvolution, ecological domains and inferred pathway footprints were obtained from only three sections (one tumor and two normal). The corrected Figure 5D eliminates technical columns mistakenly included in the pathway heatmap. No formal statistical link between the Mac_Scissor+ program and a spatial domain or PROGENy pathway was established; inferred epithelial communication was not restricted to independently verified malignant cells.

The principal limitations concern generalizability and molecular resolution. Survival-informed Scissor selection, downstream candidate ranking and survival testing reused the TCGA endpoint; patient-level pseudobulk testing of tumor versus adjacent normal specimens and repeated cross-validation of fixed candidate Cox models have now been completed, but neither tests the survival-informed cell state selection without outcome reuse. External prognostic validation and nested end-to-end discovery validation remain unavailable; the optimized score cutpoint did not reproduce its survival separation at the median. Pseudobulk tests assess tumor–normal macrophage expression rather than CTSD/HSPA1A/SPP1 survival effects, and HSPA1A did not reach significance in paired donor-level testing. Candidate gene C-indices were modest, and improvements beyond age, sex and stage were small. The spatial dataset included three tissue sections only. The in vitro work used a standardized PMA-differentiated THP-1 macrophage-like system exposed to HT-29-CM; it did not directly recapitulate or functionally isolate the patient-derived Mac_Scissor+ state. Functional experiments used the most effective of three screened CTSD siRNAs without a second-sequence functional replicate or rescue. Initial tumor CM exposure ELISAs did not separate input tumor-derived from macrophage-derived analyte, and additional polarized state comparisons were not performed. These limitations qualify the molecular specificity and broader translation of the findings but do not negate the reproducible directional changes observed across the recorded CTSD knockdown and CRC functional assays.

Taken together, the data prioritize a CTSD-associated macrophage program for follow-up studies and show that macrophage CTSD silencing changes the ability of later-collected macrophage-conditioned medium to support CRC cell phenotypes. They do not validate independent patient risk prediction, an M2-specific pathway, or direct extracellular CTSD causation.

## 5. Conclusions

Integrated single-cell, bulk transcriptomic and spatial analyses identified a CTSD-associated macrophage program in colorectal cancer. Patient-level macrophage pseudobulk testing confirmed tumor-associated increases in CTSD and SPP1 but not HSPA1A; repeated internal Cox cross-validation showed only modest gene-specific discrimination and minimal added predictive value beyond age, sex and stage. Crucially, CTSD silencing in HT-29-conditioned macrophage-like cells reduced the tumor-supporting activity of subsequently collected conditioned medium in CRC cell viability, invasion and colony formation assays. These functional results establish CTSD as a relevant experimental target in the macrophage-conditioned model, while external or fully nested selection-independent clinical validation and additional mechanistic studies remain necessary to establish patient-level prognostic performance and direct CTSD-mediated signaling.

## Data Availability

The public single-cell RNA-seq and spatial transcriptomic datasets analyzed in this study are available from the Gene Expression Omnibus under accession numbers GSE132465 (https://www.ncbi.nlm.nih.gov/geo/query/acc.cgi?acc=GSE132465, accessed on 13 January 2026) and GSE280313 (https://www.ncbi.nlm.nih.gov/geo/query/acc.cgi?acc=GSE280313, accessed on 25 January 2026). TCGA colorectal transcriptomic and clinical data are available through the Genomic Data Commons (https://portal.gdc.cancer.gov/, accessed on 28 January 2026). New processed pseudobulk expression summaries (33 specimens), paired DESeq2 results, the 571-case survival model analysis subset and repeated ten-fold cross-validation diagnostics and summaries are provided in the accompanying Round 2 analysis source data archive. Additional numerical experimental source data are available from the corresponding author upon reasonable request.

## Supplementary Materials

The following supporting information can be downloaded at: https://www.mdpi.com/article/10.3390/cancers18213464/s1, Figure S1, single-cell preprocessing; Figure S2, patient-level macrophage proportions; Figures S3 and S4, lineage/state marker distributions; Figure S5, single-cell MSI subgroups; Figure S6, survival cutoff sensitivity; Figure S7, TCGA MSI/mutation subsets; Figure S8, KEGG activity; Figure S9, spatial preprocessing and deconvolution; Figures S10–S12, CTSD/HSPA1A/SPP1 expression maps; Figure S13, cellular SPP1 sources; Figure S14, exploratory Cox analyses; Figures S15 and S16, representative Western blot source exposures; Figure S17, macrophage pseudobulk expression by specimen and matched patient; Figure S18, repeated ten-fold Cox cross-validation. Tables S1–S3 provide exploratory Cox estimates and individual Western blot band intensities and GAPDH-normalized ratios; Table S4 provides donor-level pseudobulk results and Table S5 provides apparent and cross-validated concordance indices.

## Abbreviations

The following abbreviations are used in this manuscript:

ANOVA	Analysis of variance
CCK-8	Cell Counting Kit-8
CM	Conditioned medium
CRC	Colorectal cancer
CTSD	Cathepsin D
ELISA	Enzyme-linked immunosorbent assay
GAPDH	Glyceraldehyde-3-phosphate dehydrogenase
GSEA	Gene set enrichment analysis
GSVA	Gene set variation analysis
HSPA1A	Heat shock protein family A (Hsp70) member 1A
NK	Natural killer
PCA	Principal component analysis
PMA	Phorbol 12-myristate 13-acetate
qPCR	Quantitative real-time polymerase chain reaction
RNA-seq	RNA sequencing
SDS-PAGE	Sodium dodecyl sulfate–polyacrylamide gel electrophoresis
SEM	Standard error of the mean
siRNA	Small interfering RNA
SPP1	Secreted phosphoprotein 1
ssGSEA	Single-sample gene set enrichment analysis
TAM	Tumor-associated macrophage
TCGA	The Cancer Genome Atlas
TNF-α	Tumor necrosis factor-α
UMAP	Uniform manifold approximation and projection
UMI	Unique molecular identifier
CPM	Counts per million
CV	Cross-validation
DESeq2	Differential expression analysis for sequence count data
RSF	Random survival forest
