# Supplementary material for: Single-Cell Definition and Functional Validation of a CTSD-Associated Macrophage State in Colorectal Cancer
Source: Cancers (Basel). 2026 Oct 27;18(21):3464. doi: 10.3390/cancers18213464 (PMC13762234; doi:10.3390/cancers18213464)
Supplement: Supplementary file 1 [file cancers-18-03464-s001.zip › cancers-4593116-supplementary.pdf]

Supplementary Materials

Single-Cell Definition and Functional Validation of a CTSD-Associated Macrophage State in Colorectal Cancer

Zhao Wang, Bin Hou, Chunyan Liu and Aiguo Meng

Figure S1

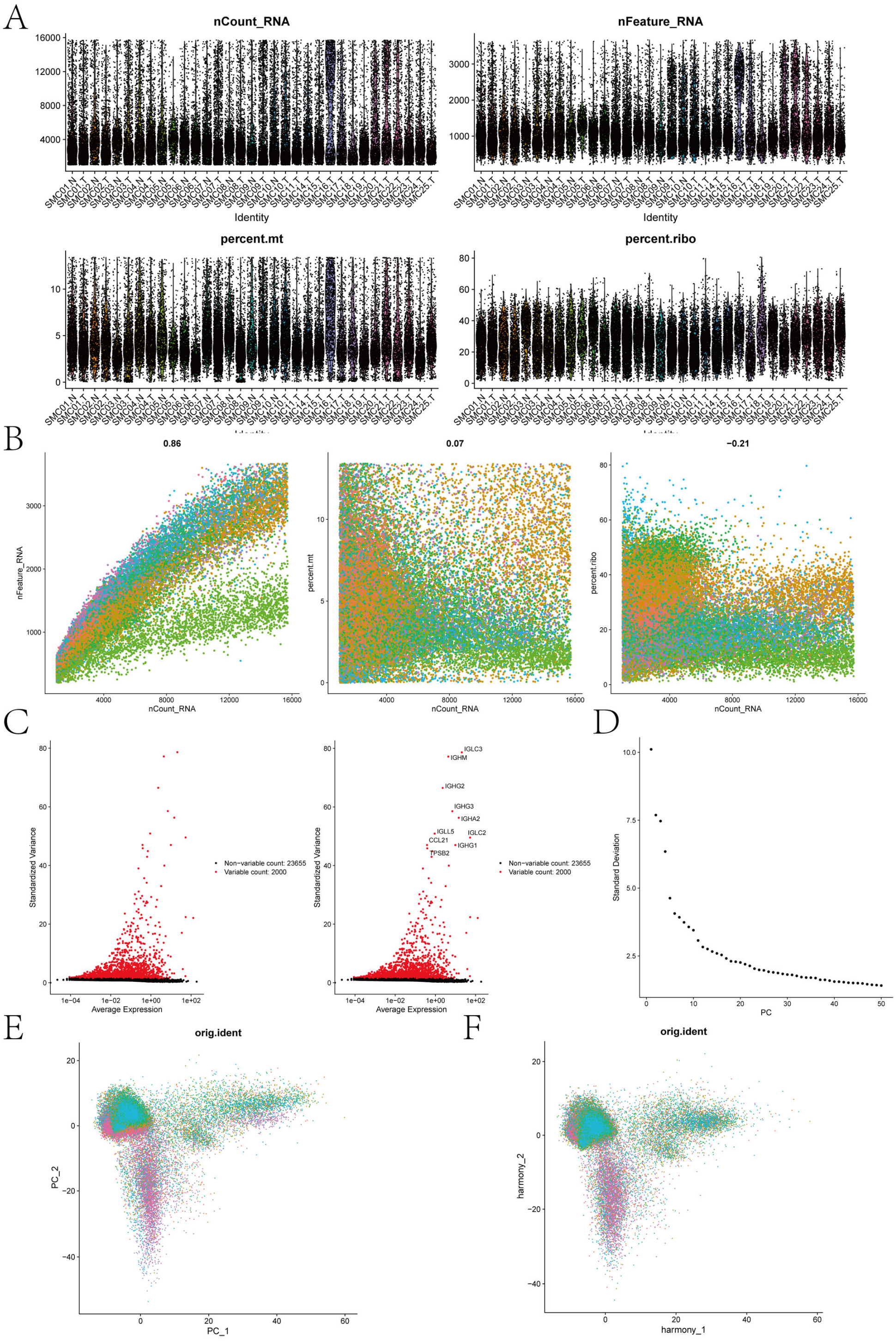

Figure S1. Quality control and preprocessing of the single-cell RNA-seq dataset. (A) Distributions of nCount_RNA, nFeature_RNA, mitochondrial-gene percentage and ribosomal-gene percentage across single-cell samples after quality-control processing. (B) Feature-scatter relationships among sequencing depth, detected features and mitochondrial or ribosomal percentages. (C) Highly variable genes selected for downstream analysis. (D) Elbow plot used to assess principal-component selection. (E,F) PCA and Harmony embeddings showing sample-level integration before downstream UMAP visualization.

Figure S2

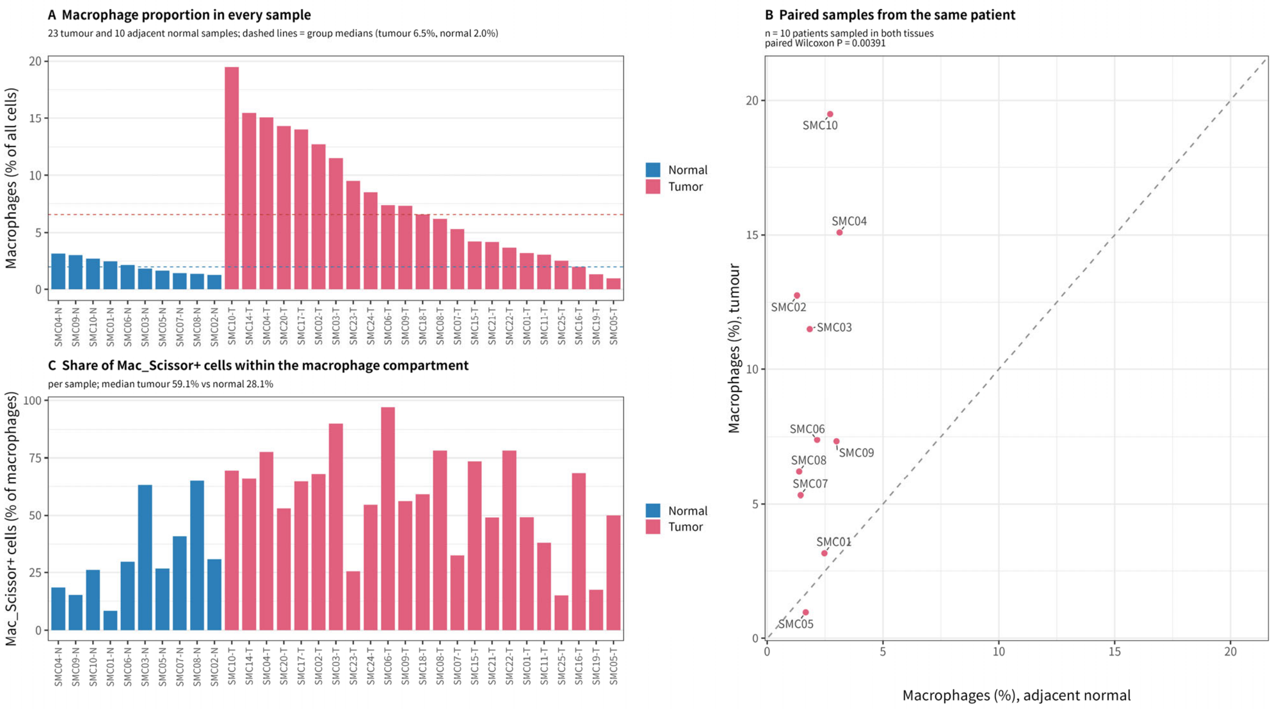

Figure S2. Patient-level macrophage representation in GSE132465. (A) Fractions in 23 tumor and 10 normal specimens; (B) ten matched tumor-normal pairs; (C) Scissor+ proportions within macrophages by specimen. Samples, rather than cells, form the unit of comparison.

Figure S3

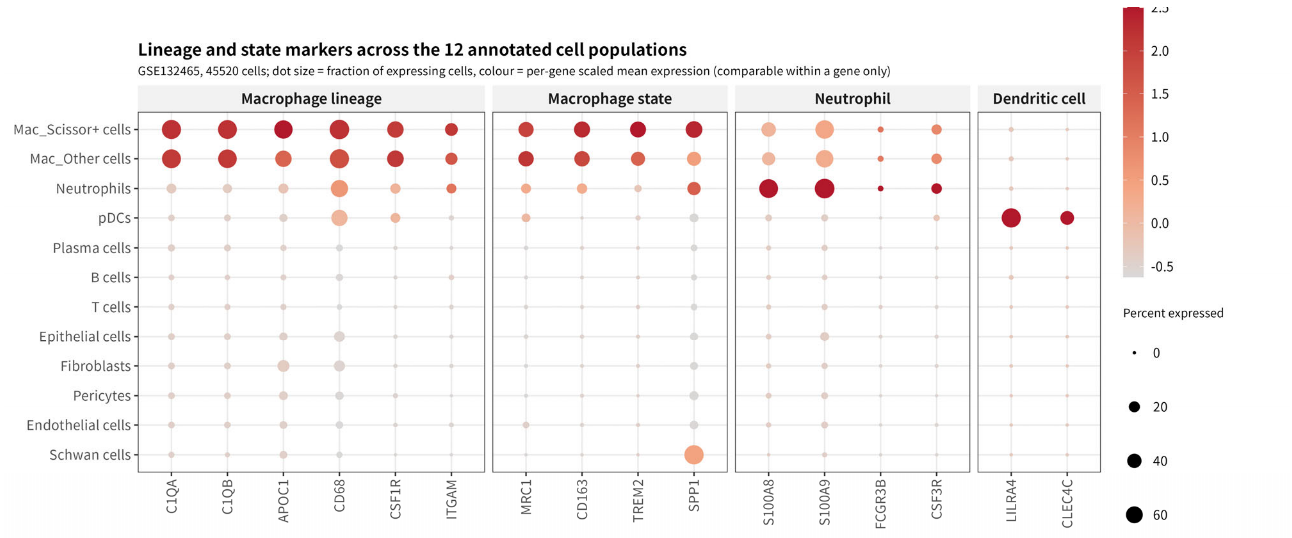

Figure S3. Lineage- and state-associated marker expression across single-cell categories. Marker panel includes C1QA, C1QB, APOC1, CD68, CSF1R, ITGAM, MRC1, CD163, TREM2, SPP1, S100A8, S100A9, FCGR3B, CSF3R, LILRA4 and CLEC4C. Dot size represents the detected fraction; relative expression color should be compared within the same gene.

Figure S4

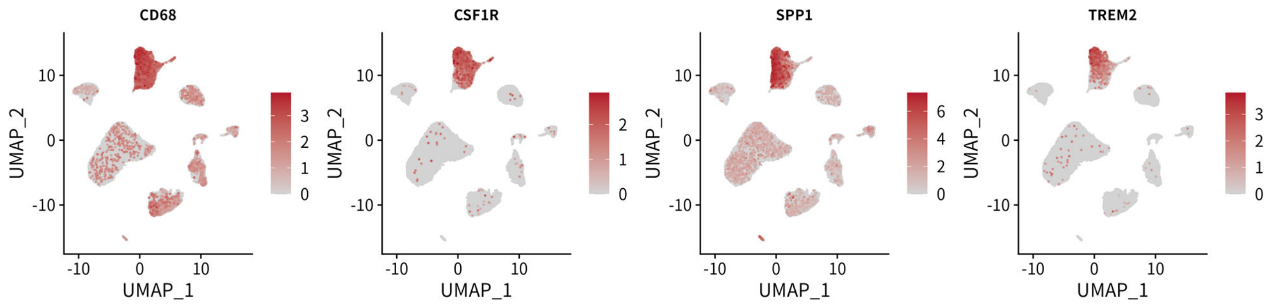

Figure S4. FeaturePlot distribution of CD68, CSF1R, SPP1 and TREM2 on the annotated single-cell UMAP. Co-expression and lineage specificity should be interpreted jointly with other markers, not from any individual gene.

Figure S5

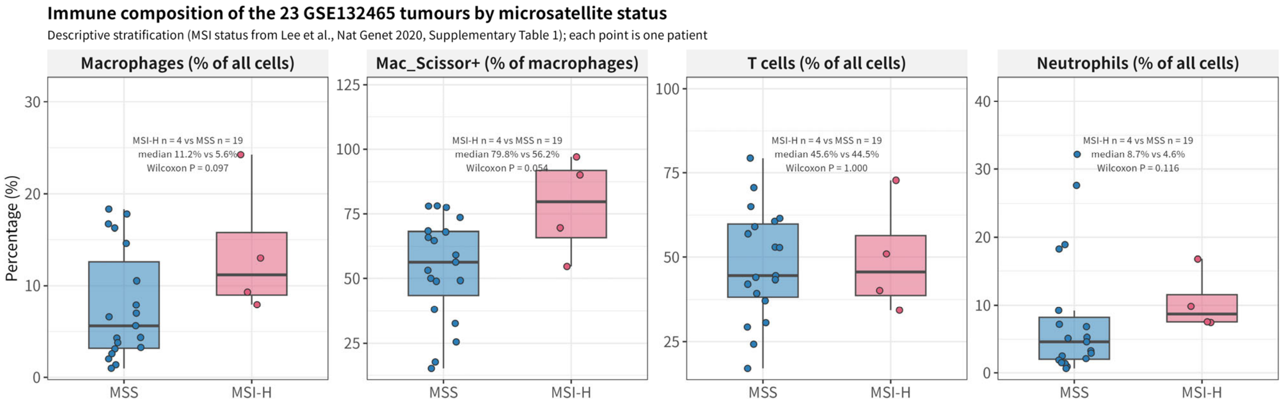

Figure S5. Single-cell cohort MSI subgroup composition. The 23 tumor samples with status annotation comprise four MSI-H and 19 MSS specimens. Displays include total macrophage, Scissor+ macrophage, T-cell and neutrophil fractions; these small subgroup analyses are descriptive.

Figure S6

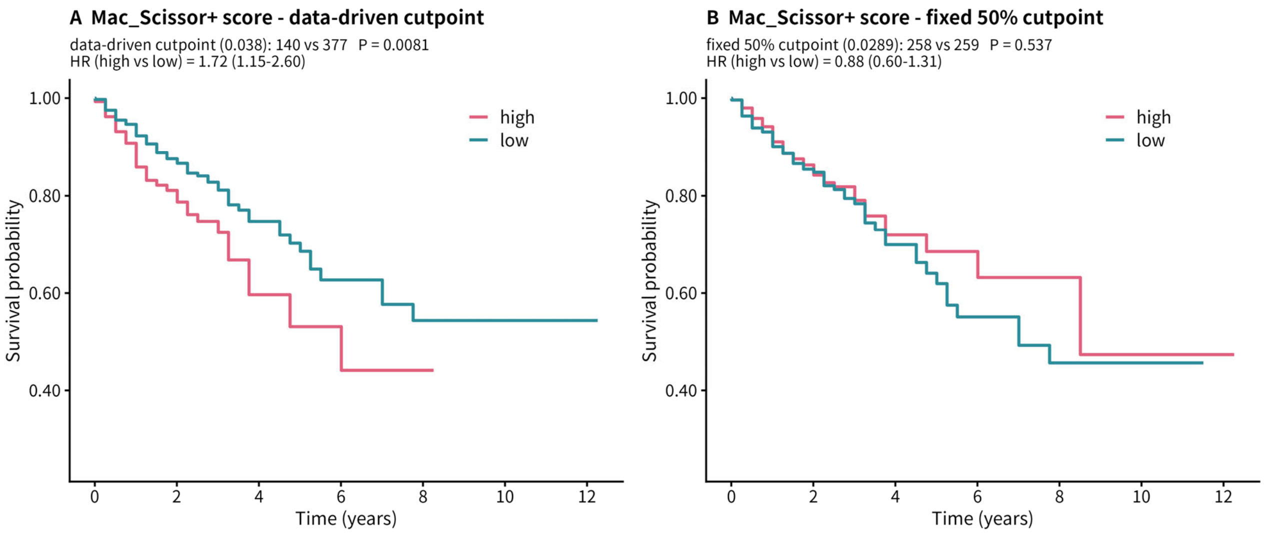

Figure S6. Survival threshold sensitivity of the Mac_Scissor+ ssGSEA program score. (A) Optimized-cutpoint groups: high 140, low 377; nominal log-rank P = 0.0081; HR 1.72 (95% CI 1.15-2.60). (B) Fixed median-cutpoint groups: high 258, low 259; P = 0.537; HR 0.88 (0.60-1.31). Both analyses reuse TCGA outcomes that informed Scissor selection and are exploratory.

Figure S7

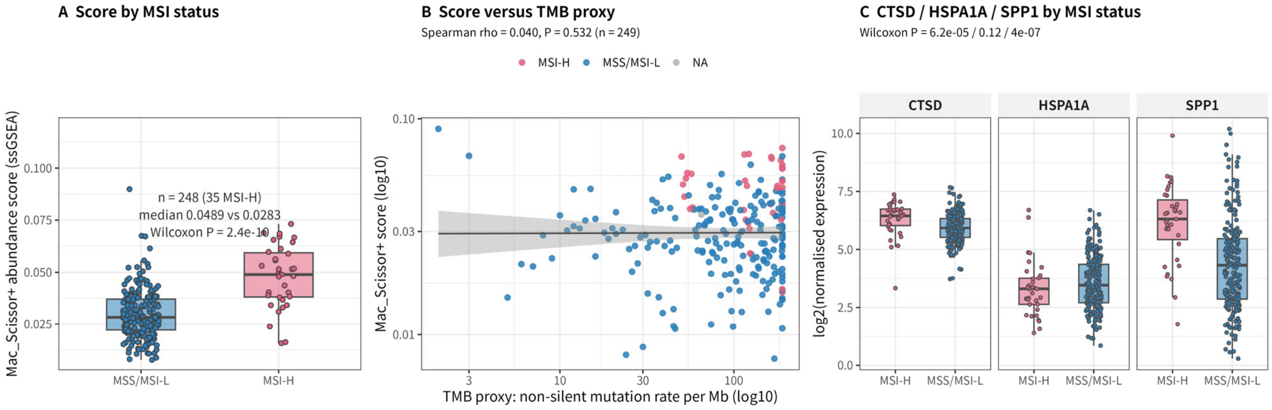

Figure S7. TCGA subset analyses by available MSI status and mutation annotation. (A) ssGSEA Mac_Scissor+ program scores by MSI grouping; (B) relation of scores to reported non-silent mutation rate; (C) candidate-gene expression across MSI groups. These analyses use only the annotated subset and do not establish independent prognostic validation.

Figure S8

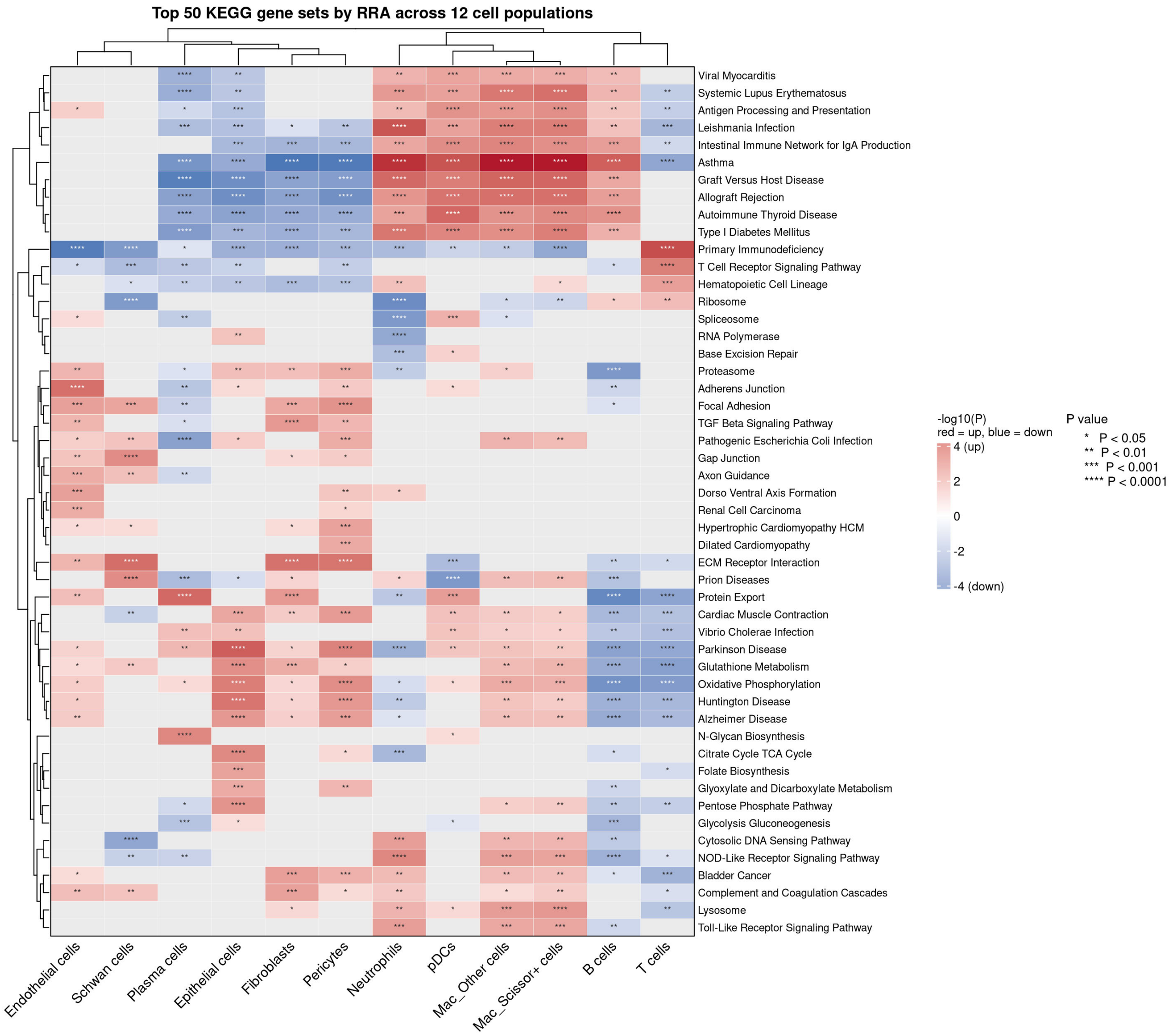

Figure S8. Complementary KEGG irGSEA analysis of the expanded single-cell categories. Pathway significance and effect direction are inferred from aggregate rank-based scoring; no functional perturbation of these pathways was performed.

Figure S9

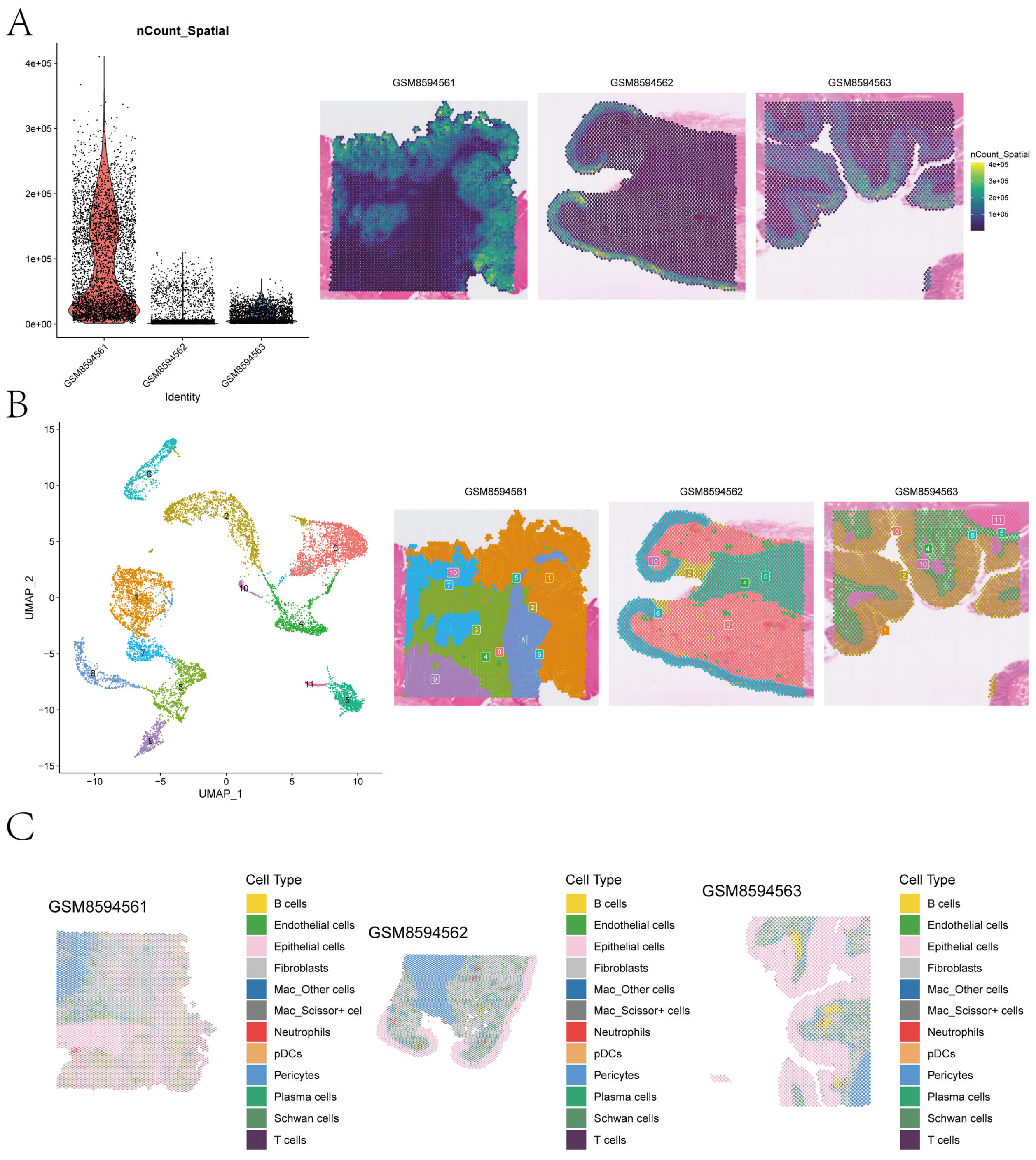

Figure S9. Spatial transcriptomic preprocessing and CARD deconvolution. (A) Spatial sequencing-depth distribution and spatial feature maps for the three colorectal spatial transcriptomic sections. (B) UMAP and spatial visualization of Louvain-defined spatial clusters. (C) CARD-based deconvolution of spatial spots using single-cell cell-type references, showing inferred cell-type composition across tissue sections.

Figure S10

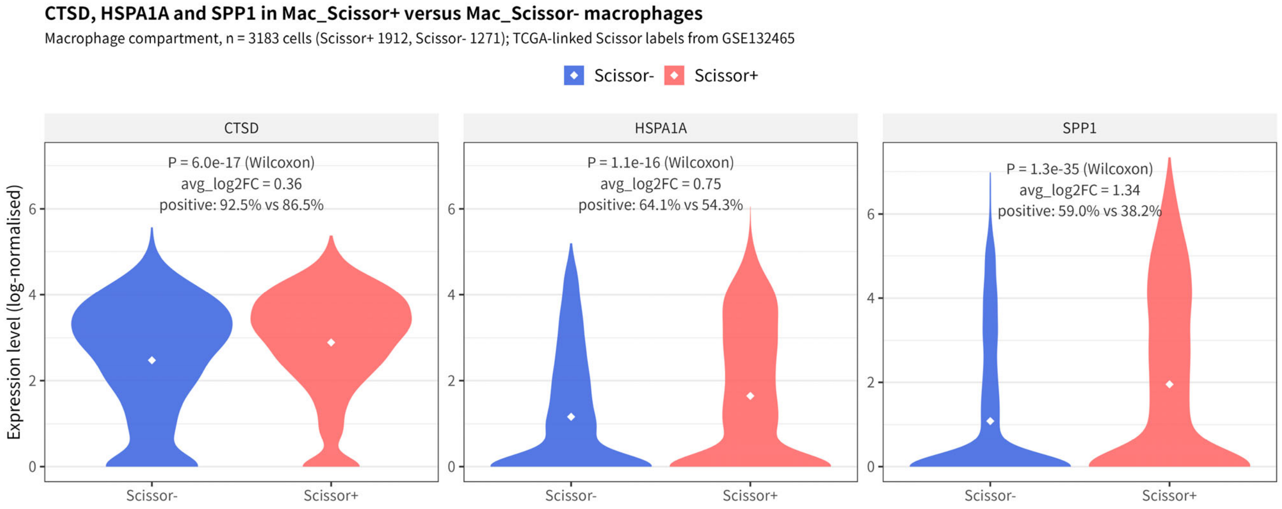

Figure S10. Violin plots of CTSD, HSPA1A and SPP1 in Scissor+ (n = 1912) versus Scissor- (n = 1271) macrophages. The plot-displayed CTSD avg_log2FC is 0.36; cell-level Wilcoxon P values and positive-cell percentages are descriptive and are not donor-adjusted.

Figure S11

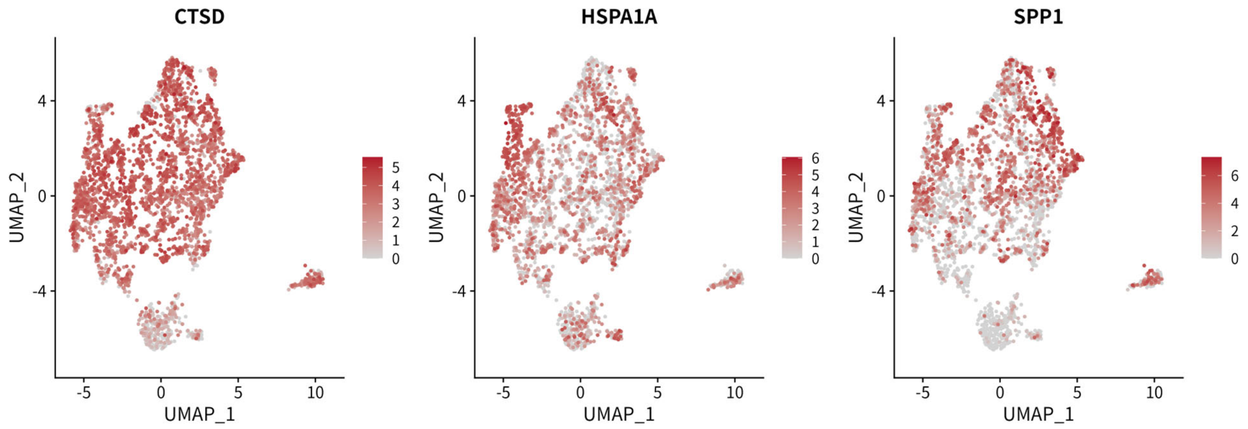

Figure S11. UMAP FeaturePlot display of CTSD, HSPA1A and SPP1 expression within the macrophage compartment.

Figure S12

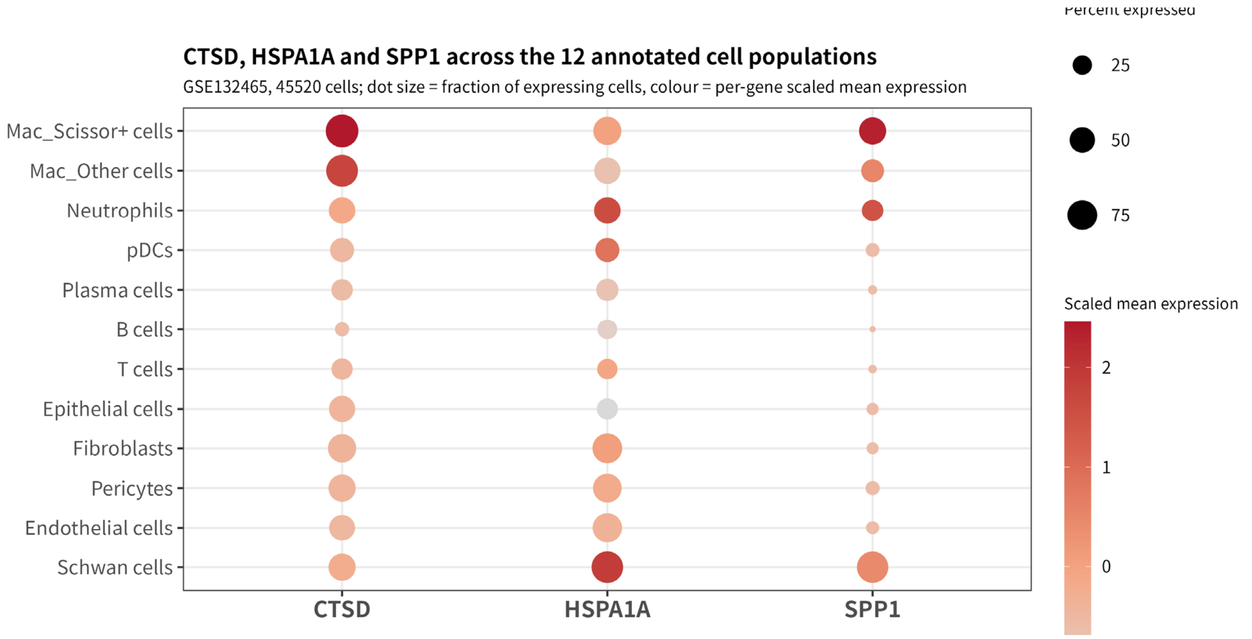

Figure S12. Dot plot of CTSD, HSPA1A and SPP1 across the expanded single-cell categories. Dot size represents percentage of cells with detected transcript and color is scaled per gene across populations.

Figure S13

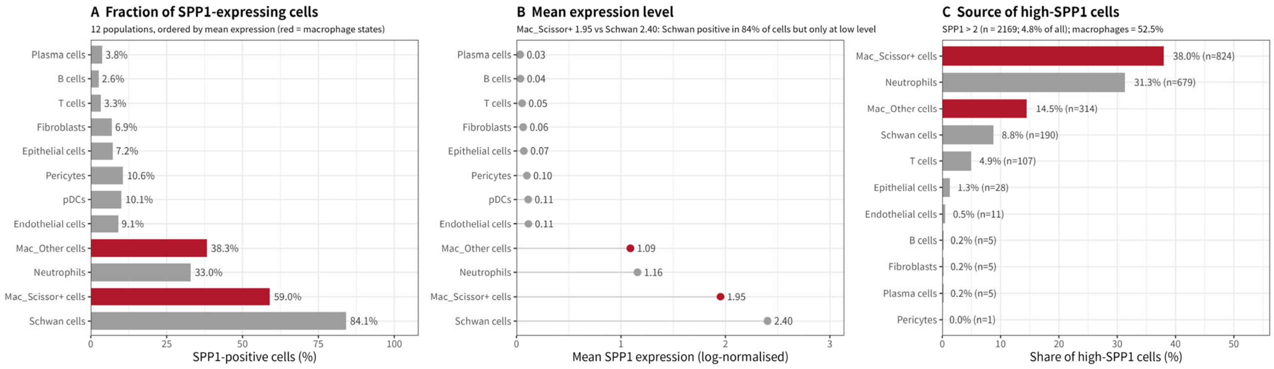

Figure S13. SPP1 expression and high-expressing cell origins across annotated single-cell populations. Different definitions of detectable versus high transcript expression yield different source fractions; macrophages were not the only SPP1-expressing cell class.

Figure S14

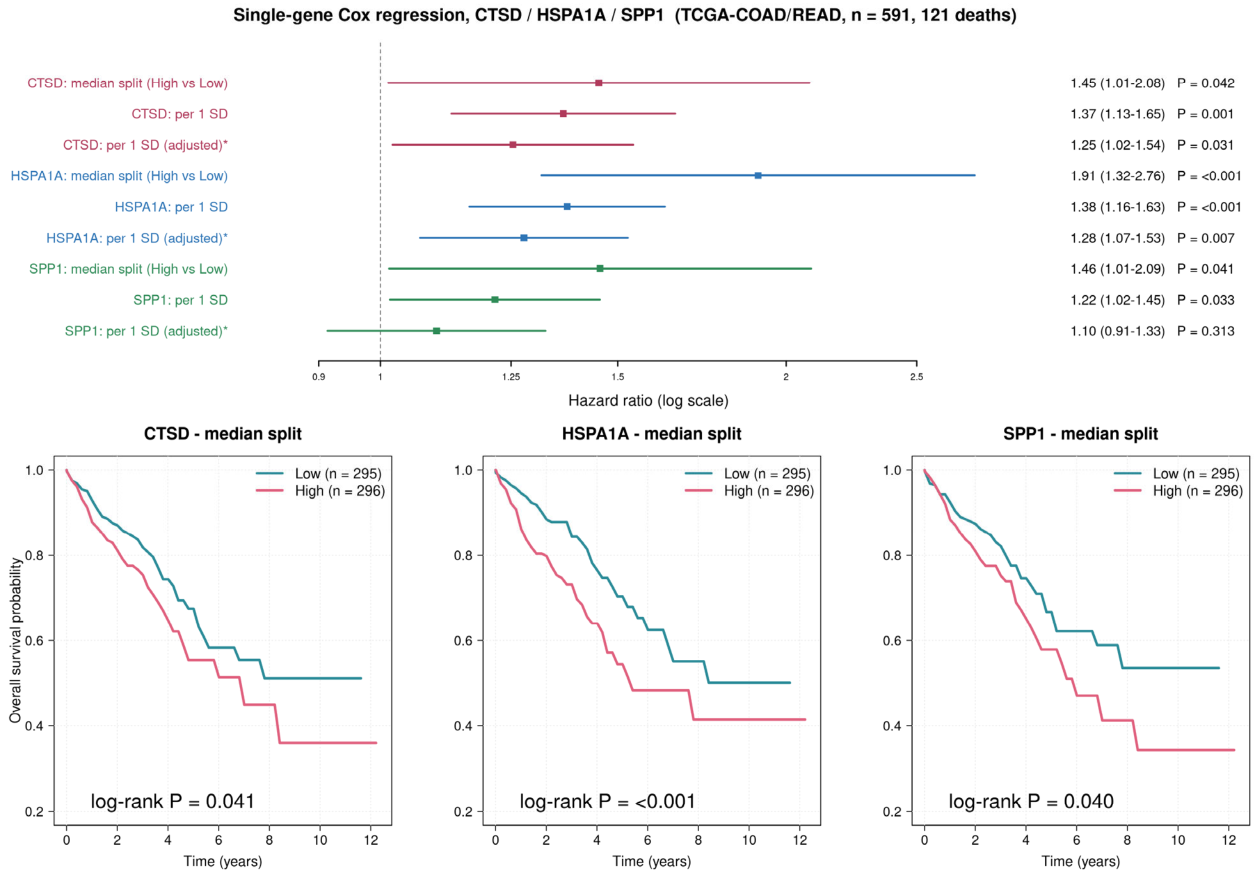

Figure S14. Gene-level exploratory Cox forest estimates and Kaplan-Meier curves. Cox estimates include median splits, continuous expression (per SD) and clinicopathologically adjusted continuous expression. Models were fit in the discovery TCGA dataset and are not externally validated.

Supplementary Table S1. Exploratory Cox Effect Sizes

Same-endpoint, internally selected (not externally validated) analyses. Univariable samples: n = 591, deaths = 121; age-, sex- and stage-adjusted complete cases: n = 571, deaths = 113. C-indices correspond to univariable per-SD expression models.

Gene	Univ. high/low HR (95% CI)	Univ. per SD HR (95% CI)	C-index	Adjusted per SD HR (95% CI)	Adj. P
CTSD	1.45 (1.01-2.08)	1.37 (1.13-1.65)	0.551	1.25 (1.02-1.54)	0.031
HSPA1A	1.91 (1.32-2.76)	1.38 (1.16-1.63)	0.596	1.28 (1.07-1.53)	0.0068
SPP1	1.46 (1.01-2.09)	1.22 (1.02-1.45)	0.543	1.10 (0.91-1.33)	0.313

Figure S15. Representative source Western blot images for Figure 9C

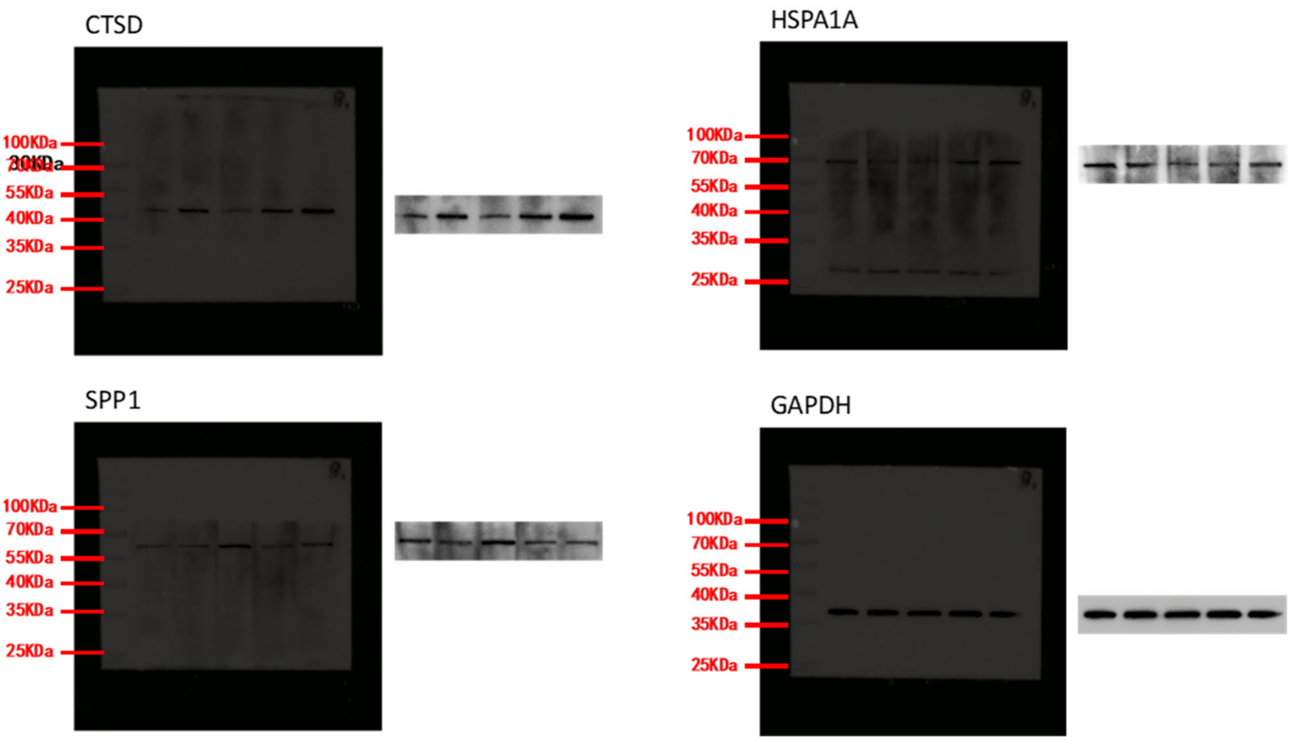

Figure S15. Available representative source exposure fields and display bands for CTSD, HSPA1A, SPP1 and GAPDH in Figure 9C. Lanes: Control, RKO-CM, HCT116-CM, SW480-CM and HT-29-CM. The six supplied TIFF files are provided separately; target and paired GAPDH measurements for the three biological repeats are tabulated in Table S2.

Table S2. Figure 9C Western blot band densitometry (three biological experiments)

Condition	Rep.	GAPDH (AU)	CTSD (AU)	CTSD/ GAPDH	SPP1 (AU)	SPP1/ GAPDH	HSPA1A (AU)	HSPA1A/ GAPDH
Control	1	2635.07	692.83	0.2629	785.90	0.2982	1135.56	0.4309
Control	2	2573.60	727.75	0.2828	788.36	0.3063	1089.59	0.4234
Control	3	2670.63	656.00	0.2456	819.07	0.3067	1166.97	0.4370
RKO-CM	1	2478.41	1370.94	0.5532	463.64	0.1871	581.30	0.2345
RKO-CM	2	2461.26	1326.53	0.5390	487.14	0.1979	570.31	0.2317
RKO-CM	3	2423.59	1398.97	0.5772	467.01	0.1927	555.13	0.2291
HCT116-CM	1	2405.22	566.80	0.2357	1030.38	0.4284	647.50	0.2692
HCT116-CM	2	2397.04	574.20	0.2395	1035.86	0.4321	625.44	0.2609
HCT116-CM	3	2402.35	542.80	0.2259	1068.33	0.4447	669.16	0.2785
SW480-CM	1	2505.05	1145.19	0.4572	367.20	0.1466	340.41	0.1359
SW480-CM	2	2501.15	1201.87	0.4805	345.94	0.1383	327.78	0.1311
SW480-CM	3	2561.69	1207.95	0.4715	350.30	0.1367	358.86	0.1401
HT29-CM	1	2120.63	1721.51	0.8118	679.45	0.3204	881.36	0.4156
HT29-CM	2	2162.17	1767.47	0.8175	647.34	0.2994	847.92	0.3922
HT29-CM	3	2168.38	1706.22	0.7869	682.87	0.3149	864.51	0.3987

Band intensities are arbitrary units (AU). For each target and replicate, ratio = target intensity / paired GAPDH intensity. Relative expression in Figure 9C was calculated by dividing each ratio by the mean ratio of the Control group. GAPDH is listed once per condition and replicate.

Figure S16. Representative source Western blot images for Figure 10C

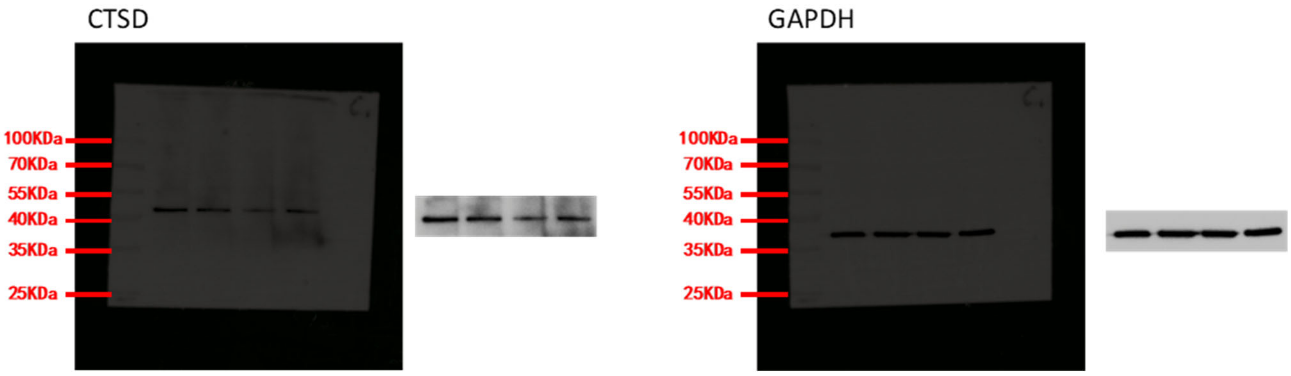

Figure S16. Available representative source exposure fields for CTSD and GAPDH in Figure 10C. Lanes: si-NC, si-CTSD1, si-CTSD2 and si-CTSD3. TIFF source images are provided separately, with densitometry for the three recorded biological repeats in Table S3.

Table S3. Figure 10C Western blot band densitometry (three biological experiments)

Condition	Rep.	CTSD (AU)	GAPDH (AU)	CTSD/GAPDH	Relative to si-NC
si-NC	1	36984.01	53507.42	0.6911	0.9920
si-NC	2	37051.20	49800.13	0.7439	1.0678
si-NC	3	37728.03	57600.60	0.6549	0.9400
si-CTSD1	1	33580.21	54694.09	0.6139	0.8812
si-CTSD1	2	35211.07	58200.06	0.6050	0.8683
si-CTSD1	3	29331.40	51100.25	0.5739	0.8238
si-CTSD2	1	19720.25	54604.03	0.3611	0.5183
si-CTSD2	2	20462.43	52200.35	0.3919	0.5626
si-CTSD2	3	19470.11	59000.39	0.3299	0.4736
si-CTSD3	1	18087.41	55667.53	0.3249	0.4663
si-CTSD3	2	18391.52	60300.03	0.3050	0.4377
si-CTSD3	3	18061.23	51900.01	0.3480	0.4994

Relative to si-NC = (CTSD/GAPDH for one replicate) / mean(CTSD/GAPDH in the si-NC group). The three repeated observations are original experimental measurements, as confirmed by the authors.

Western blot source-image documentation: Six available TIFF exposure files correspond to the representative images in Figures S15 and S16. Tables S2 and S3 contain individual target and paired GAPDH intensity readings for all three recorded biological repeats. Separate image captures for the other two repeats and independently verifiable molecular-weight annotations were not included in the supplied archive; no substitute exposures or kDa labels have been created.

Figure S17

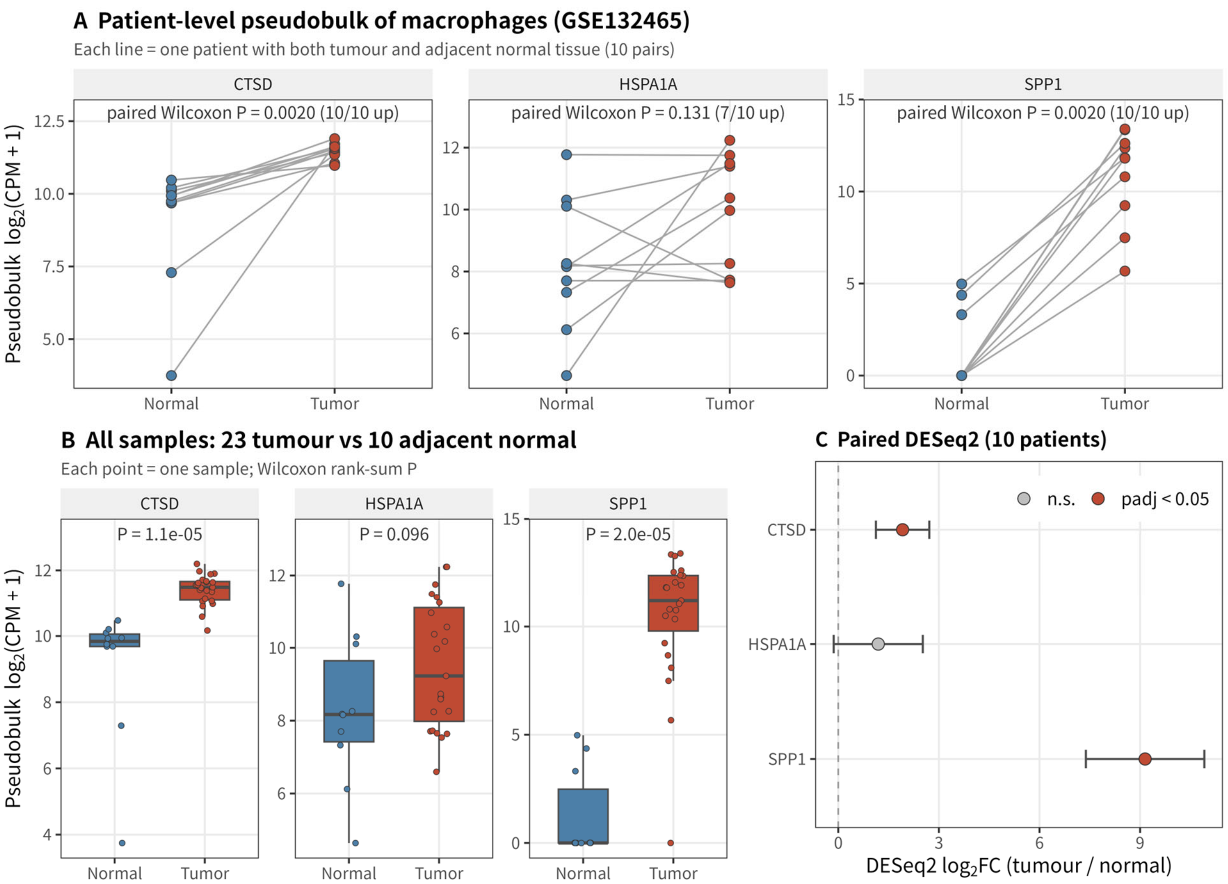

Figure S17. Patient/specimen-level macrophage pseudobulk analysis in GSE132465. (A) Matched tumor and adjacent-normal specimens from the same ten patients (exact two-sided Wilcoxon signed-rank tests; lines connect pairs). (B) All 23 tumor and 10 normal specimens, shown as sample-level observations (unpaired Wilcoxon rank-sum tests). (C) Patient-blocked paired DESeq2 analysis of ten matched pairs (log2 fold change ± approximate 95% Wald confidence interval; Benjamini–Hochberg-adjusted P values). Raw macrophage UMI counts were summed within tissue specimens, then CPM-normalized and log2(CPM + 1)-transformed for plotting. CTSD and SPP1 increased in paired tumor specimens, whereas HSPA1A did not show a statistically significant paired difference. Tumor–normal expression comparisons do not provide independent prognostic validation.

Supplementary Table S4. Macrophage pseudobulk expression validation across GSE132465 tissue specimens.

Gene	Tumor median log2(CPM+1)	Normal median log2(CPM+1)	Pairs increasing	Paired P	DESeq2 log2FC	DESeq2 padj
CTSD	11.49	9.84	10/10	0.00195	1.92	6.14×10^{-5}
HSPA1A	9.23	8.17	7/10	0.131	1.19	0.246
SPP1	11.20	0.00	10/10	0.00195	9.15	5.85×10^{-21}

Data: 23 tumor specimens and 10 adjacent normal specimens, from 23 patients; 10 patients had paired specimens. Paired tests are exact Wilcoxon signed-rank tests; DESeq2 is based on 20 matched raw-count pseudobulk samples with the design ~ patient + tissue. The normal-group median for SPP1 was 0 CPM, so a finite group-median fold change should not be reported. Unpaired P values for CTSD, HSPA1A and SPP1 were 1.05×10^{-5}, 0.0959 and 2.04×10^{-5}, respectively.

Figure S18

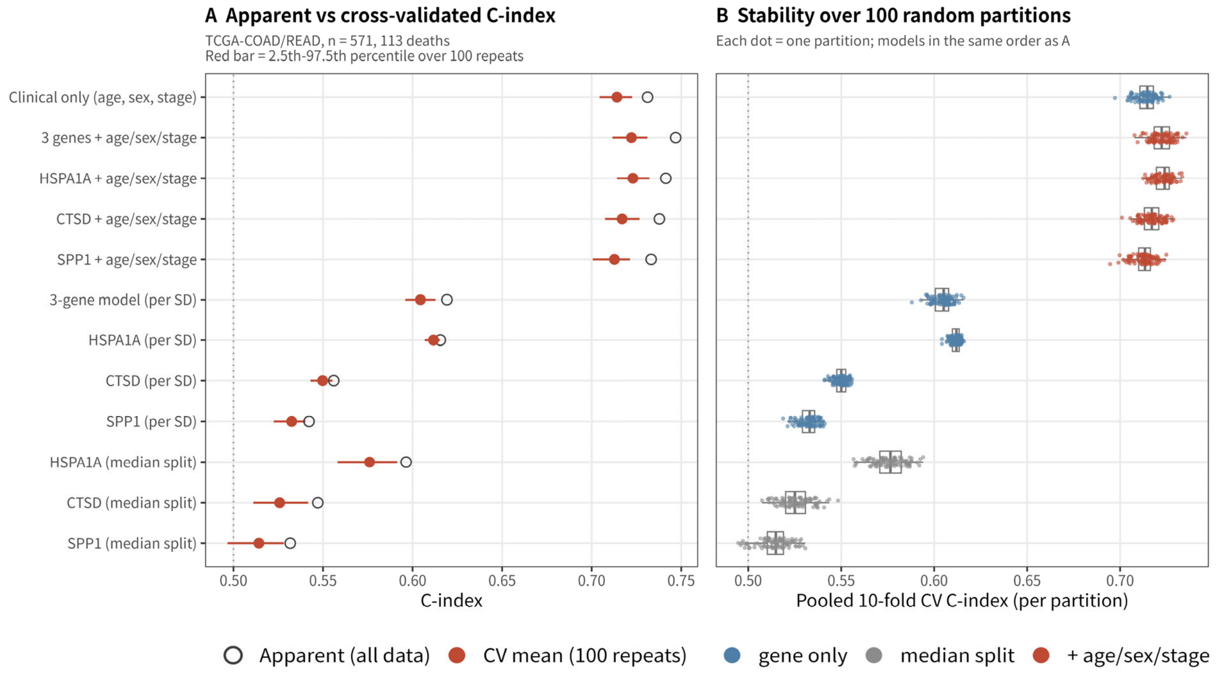

Figure S18. Apparent and cross-validated Cox-model concordance in TCGA colorectal cancer. All models used n = 571 complete cases (113 deaths). Ten-fold cross-validation stratified by event status was repeated for 100 randomly generated partitions. (A) Apparent full-data C-index (open symbols) versus the mean pooled held-out C-index (solid symbols), with the 2.5th–97.5th percentiles across the 100 partitions. (B) Each dot denotes the pooled C-index from one partition. These percentiles quantify partition variability, not uncertainty across independent cohorts. The cross-validation does not repeat phenotype-informed Scissor/gene-selection steps and thus is not selection-independent prognostic validation.

Supplementary Table S5. Apparent and 100 × ten-fold cross-validated Harrell C-index for 12 prespecified Cox models.

Model	Apparent C	CV mean C	CV SD	2.5th–97.5th percentile	Optimism
CTSD (per SD)	0.5560	0.5498	0.0034	0.5430–0.5553	0.0061
HSPA1A (per SD)	0.6155	0.6117	0.0025	0.6068–0.6152	0.0039
SPP1 (per SD)	0.5422	0.5325	0.0049	0.5225–0.5402	0.0097
Three genes (per SD)	0.6192	0.6044	0.0050	0.5959–0.6128	0.0149
CTSD + clinical	0.7378	0.7170	0.0055	0.7075–0.7267	0.0208
HSPA1A + clinical	0.7414	0.7230	0.0047	0.7141–0.7322	0.0183
SPP1 + clinical	0.7332	0.7127	0.0056	0.7006–0.7214	0.0205
Three genes + clinical	0.7469	0.7222	0.0056	0.7116–0.7311	0.0247
Clinical only (reference)	0.7312	0.7141	0.0053	0.7044–0.7226	0.0172
CTSD (median split)	0.5471	0.5258	0.0081	0.5111–0.5417	0.0213
HSPA1A (median split)	0.5964	0.5760	0.0083	0.5581–0.5914	0.0203
SPP1 (median split)	0.5317	0.5142	0.0081	0.4967–0.5281	0.0175

Clinical variables comprise age, sex and pathologic stage. Continuous genes were modeled per SD of expression; median-split cutpoints were selected within training data. CV values represent the mean of 100 repeat-specific pooled held-out C-indices. SD and percentiles describe variation due to fold assignment. Optimism = apparent full-data C-index minus mean pooled cross-validated C-index. One of 12,000 model fits produced a convergence warning; the sensitivity summary supplied with this revision found no change in the displayed mean pooled concordance values to four decimal places after excluding that fold.
